# Process-Driven and Flow-Based Processing of Industrial Sensor Data

**DOI:** 10.3390/s20185245

**Published:** 2020-09-14

**Authors:** Klaus Kammerer, Rüdiger Pryss, Burkhard Hoppenstedt, Kevin Sommer, Manfred Reichert

**Affiliations:** 1 Institute of Databases and Information Systems, University of Ulm, 89081 Ulm, Germany; burkhard.hoppenstedt@uni-ulm.de (B.H.); manfred.reichert@uni-ulm.de (M.R.); 2 Institute of Clinical Epidemiology and Biometry, University of Würzburg, 97080 Würzburg, Germany; ruediger.pryss@uni-wuerzburg.de; 3 Uhlmann Pac-Systeme GmbH & Co. KG, 88471 Laupheim, Germany; sommer.k@uhlmann.de

**Keywords:** data stream processing, cyber-physical systems, processing pipeline, sensor networks

## Abstract

For machine manufacturing companies, besides the production of high quality and reliable machines, requirements have emerged to maintain machine-related aspects through digital services. The development of such services in the field of the Industrial Internet of Things (IIoT) is dealing with solutions such as effective condition monitoring and predictive maintenance. However, appropriate data sources are needed on which digital services can be technically based. As many powerful and cheap sensors have been introduced over the last years, their integration into complex machines is promising for developing digital services for various scenarios. It is apparent that for components handling recorded data of these sensors they must usually deal with large amounts of data. In particular, the labeling of raw sensor data must be furthered by a technical solution. To deal with these data handling challenges in a generic way, a sensor processing pipeline (SPP) was developed, which provides effective methods to capture, process, store, and visualize raw sensor data based on a processing chain. Based on the example of a machine manufacturing company, the SPP approach is presented in this work. For the company involved, the approach has revealed promising results.

## 1. Introduction

In the context of the Industrial Internet of Things (IIoT), the advent of condition monitoring, predictive maintenance, and continuous improvements has influenced many technical solutions and furthered new technical developments [1,2]. Among others, one commonality of these approaches is that they rely on proper data sources, which are provided by machine sensors and their recorded data. As manufacturing machines normally comprise a multitude of sensors, one major challenge constitutes the feeding procedure of massive sensor data to downstream processing components. Available sensors must detect different sensor values to a high standard and regardless of the sensing purpose. For example, the detection of the temperature of a forming station is different to the temperature detection of a compacting station since their sensors generate data in different data formats; i.e., a conversion into standardized data formats becomes necessary. As another challenge, some sensors record data in an equidistant manner, while other sensor data values can only be recorded in a non-equidistant manner; i.e., based on cycles. Moreover, sensor data streams often pose (1) gaps or unexpected endings and (2) different sampling rates. Besides the discussed sensor differences, sensor data are typically delivered from sensor subsystems (e.g., a programmable logic controller, PLC) and continuously streamed to subsequent processing components, which must then cope with massive amounts of data. Therefore, it is important to perform the required processing tasks of sensor data in parallel. However, when utilizing parallel computing techniques in this context, time synchronization challenges must be considered. For example, if data from two different sensor signals have to be regarded for an analysis, an exact time-synchronization between these two signals must be ensured. Therefore, digital signal processing solutions are heavily used in manufacturing settings, as described here.

**Figure 1:**
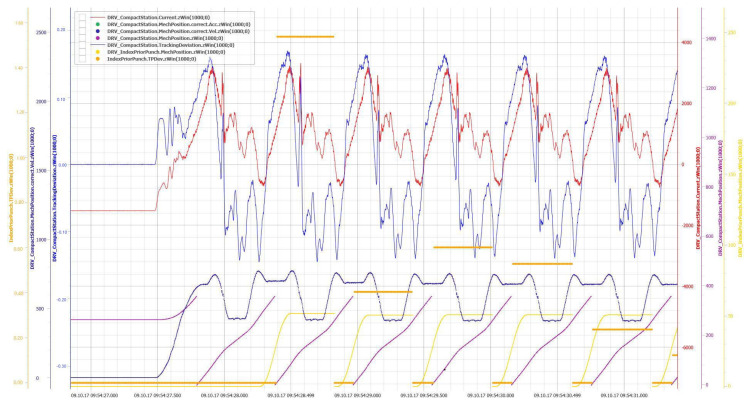
Signals of a compacting station.

To tackle the aforementioned challenges, a sensor processing pipeline (SPP) is proposed, which provides solutions for capturing, processing, storing, and visualizing raw sensor data in a continuous processing pipeline. The latter is specified as a directed graph and describes the processing flow based on processing nodes, which are able to execute different signal data processing tasks. The SPP is able to correlate different data streams. For example, the virtual position of a compacting station of a pharmaceutical packaging machine can be derived by the correlation of a time date with a power signal. Figure 1 shows an example of different sensor data values that were gathered from a motor of the above mentioned compacting station of a pharmaceutical packaging machine. Note that the diagram was generated by a self-developed visualization tool. The diagram comprises signals for the power consumption (“Current.zWin”) and the positions (“mechPosition.*”) of compacting parts. Based on the power consumption values, the exact compacting process was derived by comparing them with the current positions of the compacting parts (“MechPosition.zWin”). Importantly, the signals were correlated by a windowing algorithm that was developed in the context of the SPP. It generates and processes the compacting station signal “TPDev.zWin” in a way that the amount of required signal samples to describe the compacting process is reduced by 98%. In the following, the SPP, its main technical contributions, and the prototypical implementation are shown along the example of a pharmaceutical packaging machine line.

Production machines may consist of many distributed control systems. The latter, in turn, can operate with different time bases (see Section 2.2.1). In the presented application scenario, sensor data are provided with absolute and relative timestamps, depending on the data source. In order to process sensor data with different time bases, the timestamps must either be converted into a uniform format, or synchronization points between time bases must be defined, and the processing pipeline must support different time bases. As for the case of programmable logic controllers (PLCs), relative timestamps such as the timestamp counter (TSC; see Section 2.2.1) count the number of cycles performed by the PLC processor. Note that the transferred timestamps may not have strictly monotonous values after a system restart or when the processor’s clock is changed, and the time interval between two counting units may vary. This behavior occurs frequently and requires a continuous time synchronization mechanism. Since the definition of time bases is very application-specific and usually a large number of different developers are involved, a time synchronization schema between a PLC and a processing pipeline can be used to implement the definition of time synchronization among PLC developers, while at the same time the configuration of the processing pipeline can be automated. The presented SPP allows the use of generic time bases in which both the counting method and the time interval between a time unit can be defined. Processing nodes (e.g., to calculate the average value of a signal over time) can thus be implemented on a generic time base and do not have to be re-implemented for each time base (see Section 5.2.1). For time synchronization, the binary telemetry transport model (BTTM) was developed, which allows the synchronization of sensor data with absolute and relative time bases (see Section 5.1.1).

Production machines usually comprise hundreds of sensors (see Section 2.1). Thus, when processing sensor data from a production machine, the number of different data streams can be considerable and can quickly comprise several hundred individual data streams. Since the transferred sensor data changes depend on the state of a production machine, and therefore, the processing pipeline has to be configured to perform target-oriented analyses, it is useful to be able to group data streams logically. Many modern data stream analysis frameworks allow grouping of data streams, but they often lack the ability to define individual configurations for each group (e.g., the definition of shared triggers for generating windows). The presented SPP can logically group data streams and process data streams with different time bases within a group.

Production machines consist of a large number of components whose packaging speed (for example, the feed speed of raw production material) is synchronized by means of logical clocks. Since a change of the packaging speed does not change the sampling rate of the sensor data, the windowing of a processing pipeline must be adaptable on the basis of an external signal (e.g., the current clock speed as a virtual sensor signal to indicate the packaging speed; see Section 2.3.1). For example, a rotation signal generator of a motor delivers 600 pulses per second, i.e., 180,000 pulses per minute at 300 cycles per minute. If the packaging speed is increased, the motor rotates correspondingly faster, but the number of pulses generated is constant due to the fixed sampling rate. To be able to compare sensor data at different cycle speeds, the windowing must be adjusted accordingly, so that it is possible, for example, to define exactly one window per cycle, regardless of the production speed at which the production machine is running.

To the best of our knowledge, there is no stream processing framework that is able to define triggers for multiple data stream window generators. For better understanding the existing windowing configuration of a data stream, Apache Storm and Apache Flink are explained in the following.

Apache Storm has two basic processing methods: Core Storm and the high-level abstraction layer Trident, which was developed specifically for real-time applications based on Storm. Trident allows the definition of so-called Trident topologies, which each represent a directed graph with transformation functions. These topologies are applied at runtime to the existing Storm concepts, such as spouts as data stream sources or bolts as processing units within a processing chain. The window generation in Apache Storm can be configured for a given data stream by calling the respective window method. Therefore, the method must be called for each data stream individually. Listing 1 shows an example of the configuration of a tumbling window on a stream of “sentences,” which is triggered every 20 “word” values.

Listing 1: Apache Storm definition of a stream with count window.

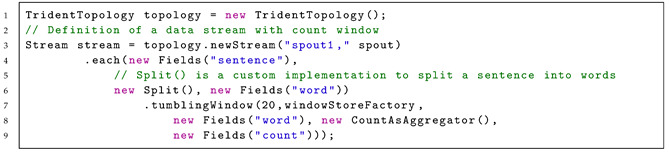



Apache Flink allows the implementation of a custom trigger, where the trigger for a session window can be defined based on the elements of a data stream. Listing 2 shows an example of a global window definition, where a new window is created if the value of an element is above a defined threshold value.

Listing 2: Apache Flink definition of a stream with global window.

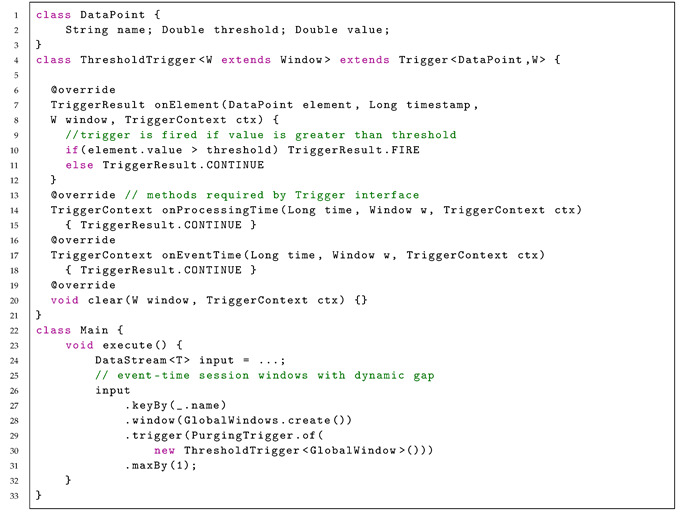



Common to both examples is the fact that for the evaluation of the windowing trigger time, only elements within the data stream to be windowed can be analyzed (see Lines 7–9 in Listing 1, and Lines 7–10 in Listing 2). In contrast, the presented SPP allows the definition of a trigger based on a data stream, which, in turn, controls the window generation of other data streams.

Furthermore, for the analysis of sensor data of a production machine, the machine cycle has to be considered for all processing steps; i.e., the window generation has to be synchronized for all data streams depending on the machine cycle. Due to the large number of data streams that, for example, a pharmaceutical packaging machine can transmit, it is advisable to define a single windowing trigger for better maintainability and to be able to reuse it across all data streams. Apache software tools (Storm, Spark, Flink), in turn, require that windowing trigger must be configured explicitly for each data stream, instead of applying a configuration that is valid for a group of data streams.

Summarizing, the framework should meet the following requirements (see Table 1 and Appendix A for framework support): (R1): Sensor data from machines are generated as data streams and should be able to be processed as such. (R2) Sensors, machines, and production runs can be configured according to customer requirements, which impact the generated data streams. For example, the installation of a compensation pendulum in a film production machine influences the forces acting on the film and consequently the sensor data that are generated. The framework should therefore support configuration variants of pipelines, windowing, and processing nodes. (R3) The framework should support equidistant and non-equidistant sensor data. (R4) Heterogeneous sensor data should be transformable in standardized data models by the framework. (R5) Gaps in sensor streams should be detected and corrected by the framework, e.g., by interpolation. (R6) The framework should be able to process data streams simultaneously. (R7) Production machines consist of a multitude of components, which, in turn, can consist of different control systems and bus systems. Each of these systems can use different time bases, for example, based on relative or absolute time stamps. The framework should therefore be able to process signals from different time bases. (R8) Production machines consist of a number of components, which, in turn, can consist of a large number of sensors. The framework should be able to group signals in order to increase maintainability and to be able to apply configurations to a set of signals. (R9) Production machines consist of a multitude of different controllers and bus systems that provide sensor data for processing. They operate on different time bases. The framework must therefore support different time bases when aggregating the data. (R10) Production processes of production machines can be mapped continuously or clocked; e.g., the machine/production speed is measured in cycles per minute. If machine speed changes, the windowing must also be adjusted based on the production cycle (a lower production speed has to result in timely longer windows). (R11) Triggers of windowing should be explicitly defined and usable for multiple streams. This improves the configuration (only one place for change), because changing the trigger configuration at 50,000 measured values would otherwise require a lot of effort. (R12) Production machines may generate large amounts of sensor data, which is hard to transfer via standard network and internet connections. Following this, the framework should process sensor data close to a production machine to immediately reduce the amount of data. Therefore, the support of commodity hardware instead of expensive server hardware is helpful.

The remainder of this work is organized as follows. Section 2 introduces the Uhlmann Pac-Systeme GmbH and Co. KG, their pharmaceutical packaging machines, relevant programmable logic controllers, and the required fundamentals for data stream and information flow processing. Section 3 introduces the context-aware process execution framework, whereas related works are discussed in Section 4. Section 5 introduces the sensor processing pipeline (SPP). The proof-of-concept implementation is demonstrated in Section 6, including (1) the evaluation of the approach with respect to its runtime aspects and (2) a discussion of its limitations. In Section 7, the obtained results are discussed, and Section 8 concludes this work and gives an outlook of possible future extensions and directions.

## 2. Background Information

In this section, background information is presented in more detail. Table 2 gives an overview of subsequently introduced aspects and their relevance for the pursued goals of the presented approach.

### 2.1. Uhlmann Pac-Systeme GmbH and Co. KG

Uhlmann Pac-Systeme GmbH and Co. KG is a mechanical engineering company headquartered in Laupheim with more than 2400 employees worldwide. Uhlmann designs, develops, and produces pharmaceutical packaging machines.

**Figure 2:**
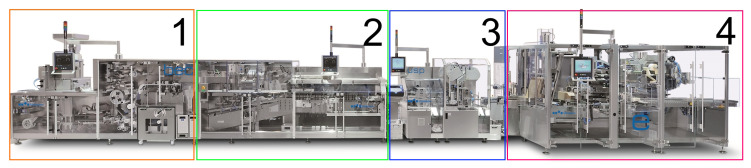
An Uhlmann pharmaceutical packaging line with its production sections.

Generally, Uhlmann machines package tablets into individual packaging units, e.g., blisters. In this process, several single machines are connected to a machine line. Figure 2 shows a production line consisting of different machines. A blister machine forms blisters with individual courts for tablets from a plastic or aluminum foil strand. Tablets are then fed and sorted into blister courts. Blisters are finally closed with a cover sheet. In production Section 2, blisters are punched out and packed into cartons, together with the folded leaflet by a cartoning machine. Production Section 3 comprises a labeling machine, in which the cartons are labeled, e.g., with a country-specific code for traceability purposes. In the final production section (Section 4), cartons are packed into boxes, which are then stacked on pallets for transportation by a case-packaging machine. Note that in addition to blisters, tablets can also be bottled with Uhlmann bottle filler machines, meaning that Uhlmann offers packaging machines for syringes and vials as well.

Legally, since all packaging machines of Uhlmann are used in the pharmaceutical industry, country-specific laws and regulations must be adhered to during the entire lifecycle of the machine. The regulations, in turn, can be mainly related to the (1) validation of the machines as a whole and to a (2) detailed documentation of all process steps that are performed during the processing of drugs. These regulations have further implications after a machine or machine line gets an approval for operation. For example, if a software component for the control or operation of a machine is changed, then further and costly validation processes become necessary.

Technically, every packaging machine continuously delivers sensor data, which can be monitored to detect anomalies very early in order to reduce downtime costs. The capturing of sensor data, i.e., the generation of actuator signals, is mainly accomplished by programmable logic controllers. As the latter are important technical components for the prototypical implementation, relevant information is presented in the following.

### 2.2. Programmable Logic Controller

**Figure 3:**
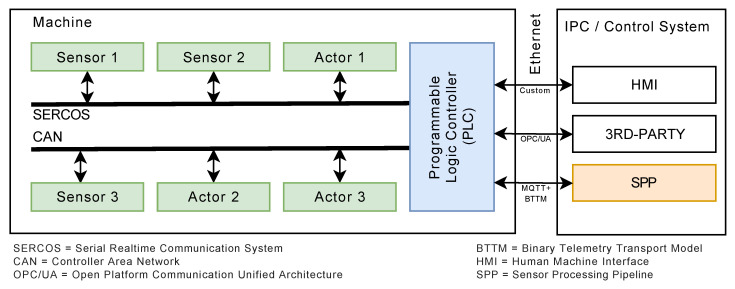
Communication schema.

A programmable logic controller (PLC) is essentially a digital computer that is used, among other purposes, for the automation of industrial electro-mechanical processes, such as the control of a machine from a factory assembly line. PLCs have input lines, to which sensors are connected, and output lines, to which actuators are connected. The latter are used to mechanically react to incoming events (see Figure 3). Input and output lines may comprise bus systems, such as the Schneider Electric CANopen [3], the Siemens Profibus [4], and the Bosch Rexroth SERCOS [5]. The applications to control machine operations are developed using different programming languages (e.g., IEC standard 61131/EN 61131), and are stored in non-volatile memory. A PLC is an example of a complex real-time system, as its output results must be produced in response to input conditions within a limited time period; otherwise, unintended operation may be the result. Typically, a PLC is connected to other information systems, such as industrial PCs (IPCs), equipped with human–machine interfaces (HMI) to configure and to control the PLC execution. Additionally, third-party components can be connected through the use of standard protocols or control systems, such as the OPC UA [6]. In the following, the binary telemetry transport model (BTTM) is presented, which was chosen for the prototypical implementation as it revealed fast and efficient transmissions of sensor data between PLCs and the other developed components of the prototype.

#### 2.2.1. Time Management in PLCs

To determine the time and the time interval between two events, different types of clocks exist in a PLC: in addition to a real-time clock (RTC), logical clocks exist [7].

The RTC measures the physical time and is typically synchronized with other clocks within a machines network via the Network Time Protocol (NTP) or the Precision Time Protocol (PTP). This allows events to be assigned to a physical timestamp that is comparable to timestamps of other computer systems. However, within the PLC, the RTC is regulated. That means, the generated RTC timestamps do not provide a linear time axis. Thus, non-equidistant timestamps may occur. As a consequence, a RTC timestamp is not suitable for applications that require a precise resolution like digital signal processing algorithms.

Logical clocks, however, do not represent the physical time. They only produce a monotonically increasing value for events, which allows for an order of events [8]. One example for a logical clock is the time stamp counter (TSC) of a PLC. This logical clock counts CPU cycles, e.g., $4.99995058798831\;\times \;10^{-10}$ s per cycle. Hence, the time difference of two events can be determined very accurately. However, the TSC cannot be used across computational boundaries for event correlation, as it resets itself each time the corresponding PLC is restarted, and thus the TSC values of two controllers are not directly comparable.

The communication between PLCs and sensors or actuators is accomplished through the utilization of different communication channels, such as a CANopen or SERCOS bus. Following the utilized bus systems, sensor measurements, which are sent to a PLC, normally take place at very different (and difficult to compare) time points due to their signal propagation or the required time for a data model conversion. For example, the desired position of a motor within the PLC must be already calculated for a later point in time to obtain the correct value after transmission to the motor control system. The actual position of a motor, when entering the PLC, has already been measured at an earlier point in time. This time difference is denoted as dead time. A negative dead time means that its value will be valid in the future, whereas a positive value will be valid if it was valid in the past. Consequently, the dead time has to be regarded when processing sensor measurements, especially when two or more sensor measurements have to be correlated.

### 2.3. Information Flow Processing

**Figure 4:**
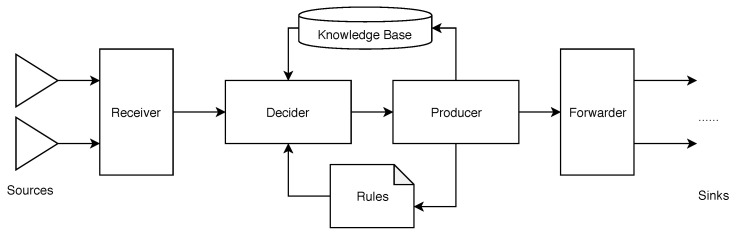
Information flow processing schema, adopted from [9].

Information flow processing (IFP) is pursued for a timely processing of large amounts of information and governed by a set of processing rules [9] (see Figure 4). For this purpose, mainly two IFP models are proposed: the data stream processing [10] and the complex event processing model [11].

The data stream processing model is used to handle streams of data, which are continuously updated. Systems that are based on data stream processing deal with continuous streams, i.e., unbounded data, and are able to handle unordered data that vary in time and size. Typically, stream data elements are processed once after arrival, followed by the processing of continuous queries. The latter are processed either periodically or continuously.

The complex event processing (CEP) model, in turn, associates semantics with data that are processed. Events are notifications denoting changes that have occurred in a sensing environment. CEP systems filter and aggregate “simple” events to higher events, i.e., being able to detect complex patterns in divergent event streams. CEP systems are based on the message-oriented interaction paradigm “publish–subscribe.” Usually, multiple single events are typically aggregated to events with higher levels. For example, the events “machine currently dropped to zero Ampere” and “machine temperature rises dramatically” can be aggregated to a higher-level event denoted with “machine overload event.”

IFP systems usually encompass the following components: one or more receivers, a decider, a producer, and forwarders. The information flow first arrives at a receiver, which acts as a multiplexer (i.e., data from multiple sources is streamlined into a continuous IFP system-internal stream) and as a mapper (i.e., data represented by a foreign data information model and received with a specified data transfer protocol are translated into a IFP-internal data information model). Following this, the processing pipeline is performed based on its processing rules. Rules determine how information blocks are managed, filtered, transformed, processed, and forwarded. This is accomplished by decider components. The latter are responsible for bounded and unbounded window generations. The way how the latter feature is actually implemented can also be regarded as a distinction criterion between different IFP systems.

Furthermore, IFP systems encompass a short-term and a long-term memory. The short-term memory acts as the storage for intermediate results that are generated within the processing pipeline. In turn, the long-term memory, i.e., a knowledge base, provides contextual information from other data sources and stores results from the IFP pipeline for further usage (e.g., for other IFP pipelines). The producer component executes pre-defined processing rules and generates new information flow elements. These are finally sent to "sinks" by the forwarder component. A forwarder component thereby acts as a demultiplexer and a mapper.

#### 2.3.1. Data Stream Windows

To process continuous data streams, evaluation windows are used. These windows divide an infinite data stream into bounded windows. Subsequently, algorithms for pattern recognition are usually executed on these windows. There exist two different types of windows: landmark windows start from a fixed landmark within the stream or take the whole stream into account (global windows), by making sliding windows’ start and end points move around the data stream by a given sliding size. The window size can be defined in different ways: time windows comprise a fixed duration. For example, all events of the last 30 s are combined into one window. In contrast, count windows comprise a fixed number of events. Finally, session windows are created based on a session key. A session key is valid for a dynamic amount of time and is defined based on a session start and end event.

**Figure 5:**
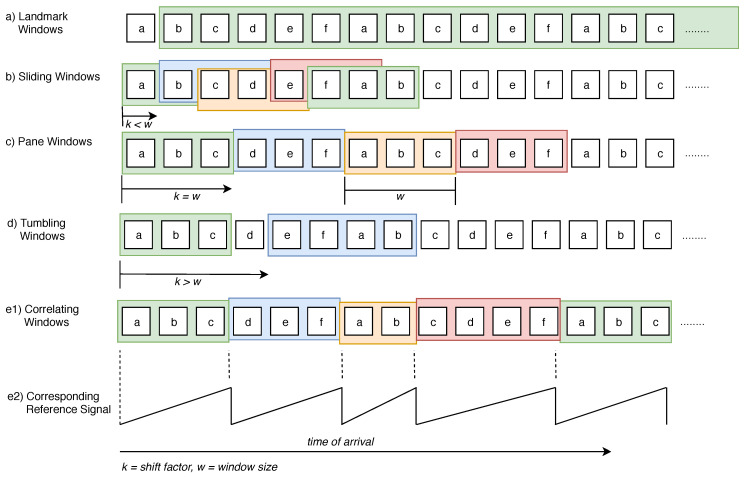
Window types and sliding window concept.

Figure 5 shows the different window types. First, landmark windows start from a fixed point in time (see Figure 5a). Next, depending on the size of the window and a shift factor *k*, three situations must be distinguished: (1) If the shift factor *k* is smaller than the window size *w*, a sliding window will be the result (see Figure 5b). (2) If both parameters are equally sized (i.e., window size and shift factor), the resulting pane windows follow each other consecutively (see Figure 5c). Finally, (3) if the shift factor *k* is larger than the window size, gaps between the windows occur (see Figure 5d). Note that the last two cases are also called tumbling windows.

For the presented approach, correlating windows are used (see Figure 5e1). The latter are a specialization of session windows. In contrast to session windows, correlating windows are triggered by one event at the beginning of a new window (see Figure 5e2). For the presented approach, correlating windows may be also based on other signals. For example, a window for a current motor signal can be generated based on the motor’s position, which, in turn, may be represented by another signal, e.g., a saw-tooth signal.

To conclude the advantages of our windowing approach, the presented approach is able to dynamically adapt to different window sizes and shift factors based on reference signals in a flexible way and with respect to the requirements of Industry 4.0 settings. For example, it enables us to capture and compare the different types of Industry 4.0 machines properly. To the best of our knowledge, such a fine-grained windowing approach has not been proposed so far in other approaches for Industry 4.0 settings.

## 3. The Sensor Processing Pipeline Approach

### 3.1. Case Study on Maintenance Processes of a Pharmaceutical Packaging Machine

**Figure 6:**
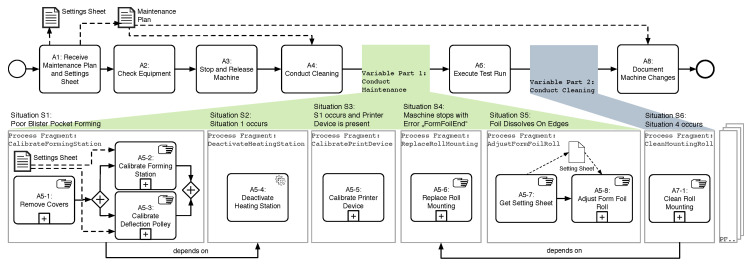
Excerpt of a machine maintenance process model (simplified).

In order to present common problems of cyber-physical processes, we conducted a study in the field of production machines [12,13]. Therefore, the maintenance processes of a pharmaceutical production machine had to be collected (see Figure 6). It was revealed that during the execution of the maintenance processes, different variants occur. Figure 6 shows an excerpt of a maintenance process. In addition to activities that must be always performed, such as “A2: Check Equipment,” there are activities which are only performed if a certain situation occurs. For example, the activities of the process fragment “CalibrateFormingStation” are only performed after execution of activity “A4: Conduct Cleaning” in case that the situation “poor blister pocket forming” occurs. This is represented in Figure 6 by the green area “Variable Part 1: Conduct Cleaning.”

### 3.2. Supporting Context-Aware Process Modeling and Execution

Based on the case study, we have elicited requirements for the support of cyber-physical systems, including (1) variability support, (2) execution and adaptation of running cyber-physical processes, and (3) traceability and logging. In order to adapt cyber-physical processes to contextual situations, context models are needed, which raise further requirements. They include (1) the modeling and representation of a context model, (2) the integration of sensor data into the context model, and (3) the evaluation of contextual situations. Further details can be found in [12].

**Figure 7:**
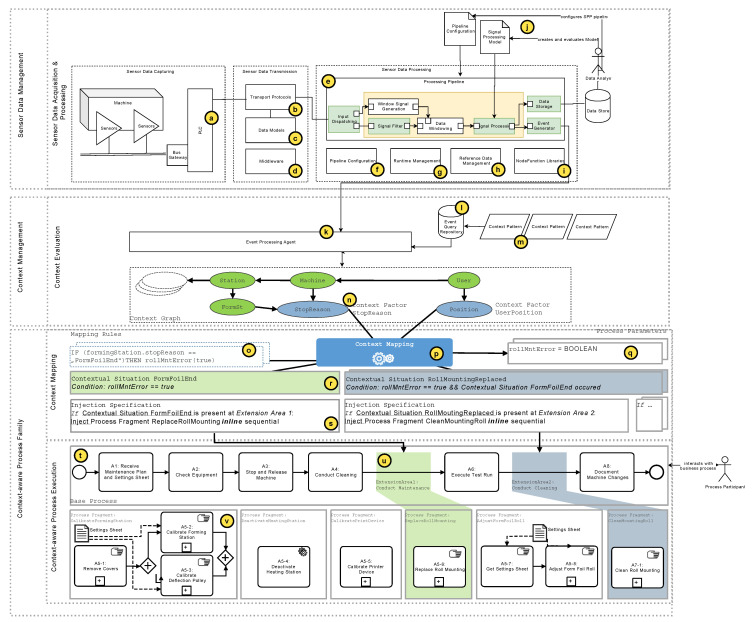
Schematic overview of context-aware process execution framework.

In order to be able to adapt cyber-physical processes based on context factors, various management components are required. In particular, the execution and runtime adaptation of cyber-physical business processes must be supported. Figure 7 gives an overview of the CaPE framework. The important parts are discussed in the following.

#### 3.2.1. Sensor Data Management

In industrial machines, sensors and actors are typically controlled by a programmable logic controller (see Figure 7a). Since collected sensor data have to be transferred to a subsequent processing component (see Figure 7e), standardized transport protocols (see Figure 7b), data models (see Figure 7c), and a transmission middleware (see Figure 7d) are required. Furthermore, a pipeline is required (see Figure 7f) to mainly cope with cyber-physical business process changes during runtime. The required algorithms for changes are managed in a separate runtime component (see Figure 7g). Obtained results can be stored in a reference data store and accessed through the processing pipeline nodes (see Figure 7h). The respective algorithms for the overall processing are grouped into node functions and stored in NodeFunction libraries (see Figure 7i). Processed sensor data can then be either stored in a database or forwarded to subsequent components. Based on this, a data analyst must define the processing pipeline configuration (see Figure 7j). Generated datasets are then analyzed and used, for example, to develop a signal processing model, which, in turn, can be implemented as node function in the Sensor Data Management component.

#### 3.2.2. Context Management

In order to manage the execution context of a cyber-physical system, events from a sensor data management component are received by an event processing agent (see Figure 7k). Events are continuously evaluated by executing queries that are stored in an event query repository (see Figure 7l). Each query can include certain context patterns to map events to entities in a context graph (see Figure 7m).

Execution contexts, in turn, can be mapped to a context graph, which is a direct acyclic graph and represents the logical structure of a cyber-physical system. Therefore, each node in a context graph has predefined context types and can be used as a basis for the concept called context-aware process family, which is introduced in the following.

#### 3.2.3. Context-Aware Process Execution Framework

Context-aware process execution (CaPE) enables the management of context-aware processes. It supports the modeling of process variants at design time and the automated, controlled adaption of processes at runtime. For this purpose, the CaPE framework uses the concept of context-aware process injections (CaPI) [14]: context-aware processes are described by a context-aware process family (CPF) (see Figure 7). The latter contains all entities that are required for modeling and executing a process that can be adapted by environmental factors: a base process model with extension areas, contextual situations based on context factors, and a set of process fragments that can be inserted into a base process model based on injection specifications.

The base process model represents a denominator of all process variants and includes all activities and decisions that are common to all process variants. Base process models are known at build time and are not changed during runtime. Areas of a control flow, on which a basic process model can differ from its variants, are characterized by extension areas. During runtime, any number of process fragments can be automatically injected into an extension area. The current context situation and injection specifications, which are based on well-defined first order logic, are used to determine the process fragments to be injected into the control flow of the base process. During injection, data flow correctness is ensured by defined process parameters. The latter are associated with dynamic external factors that influence the decision making of the process injection. These external factors can be values of context nodes of a context graph. When such a value changes, process parameters can also be changed on an event-based basis, triggering a change to one or more processes. During process fragment injection, CaPE ensures correct data flow mappings.

CaPE enables the controlled but dynamic configuration of changes of different processes at runtime. Through a multi-model approach it simplifies the reuse of process fragments in different process variants instead of requiring a highly complex process model that captures all variants. In addition, CaPE enables the mapping of contextual changes during runtime by late selection of contextual situations at specific extension areas in order to adapt a process by the correct process fragments. The automated and consistent mapping of the data flow between the injected process fragments and the underlying basic process reduces the effort for the users involved. Note that the CaPE process was implemented as a prototype to demonstrate its feasibility [15].

#### 3.2.4. Summary

The CaPE framework enables the context-aware execution and adaptation of cyber-physical processes. Business processes can be adapted based on the sensor data of a cyber-physical system. The context management ideas and execution of context-aware processes of CaPE can be found in these works [12,14,15]. On top of CaPE, it is shown in this work, how (1) sensor data can be acquired from a cyber-physical machine and (2) transmitted between the sensors acquisition component and a processing component. The latter transform sensor data into events that can be further used to maintain a context model, which, in turn, supports a context-aware process engine to enable runtime adaptations of business process.

## 4. Related Work

Stonebraker et al. describe eight requirements for real-time data processing systems [16]. As they are closely related to the presented concept, a comparison will be discussed in the following.

The first requirement (*SR1: data must remain in motion*) states that data should be processed without using caching mechanisms. Considering the concept and implementation of the SPP, there is no caching of the data and this requirement is met. The second requirement (*SR2: using StreamSQL*) states that streamed versions of the SQL should be used to formulate queries based on the streams. The reason for this is to ease the understanding of the relevant processes as they are defined in a semantically higher language like C++ or Java. This is currently not supported in the SPP. Since streams are transmitted via different, sometimes unreliable transport protocols, and depending on the application, it is necessary that the processing system can handle *SR3: errors of data transfer*. Errors, in turn, can be missing data or data being transmitted in the wrong order. It must be ensured within the processing system that no missing data results or disadvantageous waiting times occur. The SPP supports mechanisms for this requirement. The results of the processing must be *SR4: predictable and reproducible*. This requirement could be confirmed for the SPP through the various runs of the performance tests. However, this requirement cannot be considered by the SPP, as it highly depends on the concrete node implementation.

According to the requirement *SR5: linking stored and current data*, there should be the possibility to access current and already stored data. The reference data memory of the SPP offers the possibility to store comparison data and its retrieval. The sixth requirement is concerned with the *SR6: availability of the system*. This can be ensured by the SPP as every component of the SPP can be executed in parallel. The streaming system should *SR7: auto-scale* across multiple processors and computers, allowing for a better data processing. The SPP uses all available cores within a computer for processing. Since the current architecture requires that a SPP component is available for each PLC, a further scaling mechanism over several machines is not necessary. Horizontal scaling—in the case of the SPP—implicitly takes place through several independent systems. Stream processing should be done with a minimal overhead, so that *SR8: real-time responses* can be triggered. Through data processing within a process and lightweight base structures, the sensor data, as shown in the performance test, can be processed very quickly. However, the *TriggerNodes* and *AggregatorNodes* cause a delay in the data processing procedure.

Dunkel et al. describe an event-based architecture for processing sensor data based on the complex event processing paradigm [17]. In this system, the sensor data are interpreted as raw sensor events and then transferred to domain events, status events and action events. For this purpose, event processing languages (EPLs) are used. Based on this, the system offers the possibility to derive actions from raw sensor events. The SPP provides a more flexible approach to process sensor data. In addition to the aggregation and search functions for pattern recognition contained in the EPLs, it is possible to execute arbitrary algorithms for digital signal processing within SPP nodes. Results from nodes can be used to identify patterns or changes in subsequent nodes. As the outputs of the SPP are flexible, the methods described in [17] can be applied through the SPP.

Stocker et al., in turn, describe a multi-layered system that allows for the abstraction of semantic situation descriptions from sensor data and contextual information [18]. In particular, techniques from the areas complex event processing, machine learning, and Semantic Web are used. These ideas and techniques have not been tracked and implemented in the context of the SPP.

Problems of temporal synchronization of different sensors is described by Rodriguez et al. [19]. Each sensor measures data at different times, depending on the type of the sensor, and transmits the data to the processing system. For correct evaluations, it is necessary to know the exact timestamps of the data. In [19], all sensors are connected directly to a micro-controller for evaluation. Therefore, the internal Real-Time Clock (RTC) will be used to determine the timestamps of the measurements. This procedure is also used by the SPP concept. The central element here is the PLC, for which the data of the sensors and the running times of the signals are known via the provided real-time bus systems. Instead of a single RTC timestamp, the exact time stamp count (TSC) value and its end time of the signals are additionally transmitted. This information is necessary as the signals of the different sensors pose different signal propagation times to the PLC. Thus, the presented concept is an extension of the technique presented in [19].

The Message Queue Transport Protocol (MQTT) and the Advanced Message Queuing Protocol (AMQP) are application layer protocols for message-oriented middleware systems [20,21]. They can be used to transfer sensor data as well. The developed binary telemetry transport model (BTTM) is lightweight, binary-encoded, and poses less overhead. It offers specific frames for time synchronization and can be also used as a payload format for MQTT and/or AMQP, which offer error correction, session management, and security methods, such as authentication or transport encryption.

Regarding data stream processing, various related works exist [22]. Borealis is a stream processing system developed by three universities: Brown and Brandeis Universities and the MIT [23]. Borealis is based on the research projects Aurora and Medusa [24]. It has a graphical interface that allows one to graphically model the stream processing. As in KNIME or RapidMiner, individual processing components, here *boxes*, are linked to each other. However, the Borealis system is based on SQL-like, continuous queries, and has similarities to Esper and CEP. It implements eight basic operators, including one that enables windowing [23]. These operators, in turn, enable sliding windows known from CEP, or tumbling windows and their special forms. However, the concept of correlating windows does not exist. The other operations are also based on the functions known in SQL or the Continuous Query Language (CQL). Specifically, filter, groupBy, and join operations are provided that filter individual elements from streams, create groups, or merge multiple streams. Notably, only the map operation allows for a user-defined function to be applied to all tuples within a stream. Thus, the concept of nodes in the SPP is more flexible. Contextual sources and meta-information about individual streams are not modeled separately in Borealis. Thus, contextual information must be added to the individual data streams by means of union operations. Likewise, necessary meta-information must be transported within the streams. To conclude, the concepts shown for the SPP component—with respect to sensor data processing—are in line with the advantages shown for Borealis. However, Borealis includes components, such as a graphical interface or a runtime tracking, that are not yet included in the SPP, but can be easily implemented. A common concept in the field of digital signal processing is called signal flow graphs (SFG). It was firstly introduced by Claude Shannon [25]. SFGs represent a set of simultaneous linear algebraic equations, for example, to characterize and analyze sequential electronic circuits. A linear SFG consists of nodes and weighted directional edges. The nodes are the variables of the equations and the branch weights are the coefficients. Signals may only traverse a branch in the direction indicated by its arrow. The elements of a SFG can only represent operations, which are sufficient to represent constrained equations. When a signal traverses a branch in its indicated direction, the signal is multiplied by the weight of the branch. When two or more branches direct to the same node, their outputs are added.

The lightweight dataflow (LWDF) is an approach for model-based design and implementation of wireless communication and software-defined radio systems [26]. It offers a dataflow model for software defined radio (SDR), for which signal handling methods, e.g., demodulation of a signal, are implemented as software functions rather than special-purpose hardware. LWDF extends synchronous dataflow graphs, which are loosely coupled based on the concepts of signal flow graphs and consist of actors (i.e., vertices of a graph, software functions) and directed edges between these actors. The latter represent a first-in-first-out (FIFO) queue. LWDF further extends SDG with ports and an operational context for every actor, for which the operational context consists of parameters, local variables, state variables, and a reference to execution functions of actors. In contrast to the SPP, window generation is limited to fixed frequencies.

In the context of software defined radio, various implementations exist. LuaRadio defines composition blocks, which may consist of sources, sinks, and processing blocks, whose input and output ports are connected to edges (http://luaradio.io). Sources generate signals and sinks consume them (no output port), while processing blocks transform signals. LuaRadio supports asynchronous data flows based on Integer vectors. However, signals are windowed based on Int32 vectors. In contrast to the SPP, there are no dedicated time base and time stamps. Furthermore, signals are processed without having any additional contextual information, like a time stamp of the signal generation or its name, which, in turn, are supported by the SPP. Additionally, conditional flows and iterations of blocks are not supported as well.

Aldinucci et al. developed FastFlow, which is a programming framework for memory-shared multicore environments [27]. It is based on a general purpose layered architecture and supports stream applications. In this context, emitter, worker, and collector nodes allow for the definition of processing pipelines, similarly to the SPP. However, the framework focuses on generality and performance rather than usability. Following this, the SPP was developed for the purpose to process sensor data. Unlike FastFlow, the SPP simplifies data handover between different nodes by providing standard interfaces, pre-defined nodes support, for example, noise cancellation and correlating windows. FastFlow is implemented in C++ and provides lock-free and memory fence free synchronization mechanisms, like the ones used in the SPP.

Apache Flink is a framework for the distributed processing of data streams. It is based on an event-driven architecture and offers mechanisms for time handling. For example, Flink is able to handle data events that are transmitted after so-called late events. Flink can be executed in a distributed environment. Every execution environment contains different functions for different data sources, window generation, data stream transformation, and data sinks. Executions plans allow for a pipeline-based configuration, such as provided in the SPP. Different APIs enable Complex Event Processing (CEP) and analyses on graphs. Different libraries exist for Machine Learning algorithms, however, no libraries for signal processing related functions such as the reduction of noise like in the SPP exist so far, to the best of our knowledge.

Konstanz Information Miner (KNIME) is a modular environment, which enables an easy-to-use way to visually assemble and execute data pipelines [28]. It provides the simple integration of new algorithms and tools and data manipulations or visualization methods in the form of new modules or nodes. A KNIME workflow is a directed acyclic graph containing nodes, which can be freely distributed (multi-threaded). However, data to be processed by KNIME nodes have to be serialized for every node execution, which negatively affects the overall execution speed. Execution loops can be created by special loop nodes, which can communicate with each other. However, KNIME cannot process stream data; i.e., it is limited to batch processing with the goal of applying algorithms to train cluster models, regression models, trees, or support vector machines. Stream processing support for KNIME is currently under development. Trained models can be exported using the Predictive Model Markup Language (PMML) [29]. In summary, KNIME offers an environment for a batch-oriented machine learning development, while the SPP can be used to evaluate these learned models against data streams.

There are publications on various applications, which collect, analyse and forward sensor data. These can be roughly divided into two categories: those whose analyses run next to sensors and those whose data are forwarded to a remote component and analyzed there.

Kanawaday et al. describe an approach, where sensor data from a slitting machine are captured by a PLC [30]. The data are transferred to an industrial PC (IPC) via a RS485 serial bus, converted into MQTT packets, and transferred to a remote cloud service, where the data are analyzed based on AutoRegressive Integrated Moving Average (ARIMA). However, little is known about the sensor data payload format and the amount captured. Streams are not correlated with each other and no windowing and time synchronization are applied.

Canizo et al. describe a real-time analysis of wind turbine data [31]. A dataset containing alarm events is emitted by a SCADA system every 10 min to a Apache Kafka message broker via the AMQP protocol. The analysis is performed with Apache Spark, which uses the random forest algorithm to generate predictive models for the monitored wind turbine. Although the authors describe that operational data can be collected, only status data, i.e., alarms, are used for analysis. These are already available in a structured form and require only very small amounts of data.

Donovan et al. present a big data pipeline for integrating, processing, and analyzing industrial equipment data in the large [32]. A study was conducted in a manufacturing facility of the Johnson and Johnson group to observe technologies, architectures, and configurations in a real-world industrial environment. One research question concerned the development of a big data pipeline to support equipment maintenance. The presented architecture comprises distributed software agents that collect time series of industrial equipment and forward them message-oriented to a cloud service that provides a data repository. The stored time series can then be pre-processed using a processing component, and for example, be aggregated by time period. Furthermore, normalized time series are analyzed and interpreted in a batch-oriented manner. The architecture presented does not provide any further details about the concepts and technologies that can be used for pre-processing and analysis.

Schweppe et al. describe stream processing of Automotive sensor data [33]. Therefore, sensor data represented in the keyword protocol (KWP) are captured with an on-board diagnostics (OBD) interface from a standard automotive controller area network (CAN) bus. On-board processing of the data is defined as a directed acyclic graph named stream processing graph (SPG) similar to the SPP, in which processing steps are defined with nodes. The SPG supports the application of windowing as sliding and tumbling windows, however, in contrast to the SPP, correlating windows are not supported. Furthermore, the SPG does not support different time bases, since the data are already available in a standardized form with a standardized time stamp. This usually cannot be assumed for industrial production machines, since they typically consist of many components that are developed by different manufacturers without common specifications.

All the approaches presented have in common that they use much smaller sample rates of the sensors, and thus, cause a smaller amount of data. In our setting, we have sample rates of up to 1 ms, which allow much more precise analyses. For example, very small speed fluctuations can be detected, which could not be detected at higher sample rates by undersampling.

Since large production lines typically consist of several PLCs that are networked together, generated samples must be time-synchronized. The approaches above do not allow the synchronization of different physical and logical time bases without the need to adapt the implementation for each transformation step.

Furthermore, none of the approaches describe a process-oriented and unified configuration of pre-processing, analysis and forwarding tasks. Instead, software artifacts have been created that cannot be managed centrally via a uniform pipeline model, but have to be adapted individually in a time-consuming and error-prone manner when changes are made.

Some of the approaches require complex cloud architectures. However, there is a need for a lightweight component, that can be installed on a standard IPC and does not require any cluster configurations for data analyses, since the large amounts of data generated can already be analyzed and aggregated on an IPC. This circumstance can eliminate the requirement for sufficient bandwidth between the machines and an analysis running in a remote cloud and is particularly advantageous at production sites with small and unstable internet connections.

## 5. Concept Overview

**Figure 8:**
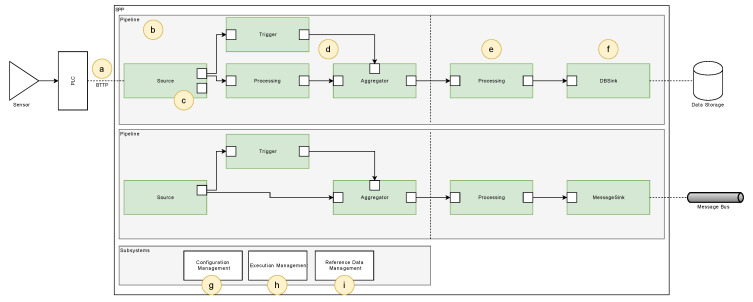
Schematic Overview of the sensor processing pipeline (SPP).

In the following, the conceived and developed sensor processing pipeline (SPP) is presented and discussed. Figure 8 shows the subsystems, components, and interfaces of the SPP.

In this section, preliminary data management aspects are discussed. First, the developed binary telemetry transport model (BTTM) is introduced (see Figure 8a), while then presenting the SPP pipeline concept (see Figure 8b–f). Furthermore, the configuration of a SPP pipeline (see Figure 8g), the execution logic (see Figure 8h), and the data management (see Figure 8i) are discussed.

### 5.1. Sensor Data Capturing

In order to be able to capture sensor data, a communication interface between sensors in question and the SPP is required. Therefore, a sender of sensor data (e.g., a PLC) and the input component of the SPP, the SourceNode, needs to negotiate on a joint communication protocol. Common IoT protocols like the Message Queue Telemetry Transport Protocol (MQTT) or the Advanced Message Queuing Protocol (AMQP) are examples that can be used in this context. For the communication procedure in general, the sensor data payload must contain (1) a unique identifier to be able to differentiate different sensor signals, (2) a system-synchronized timestamp mechanism to be able to correlate signals from multiple senders, and a well-defined data format that captures the signal data. Due to time synchronization and performance requirements pertaining to the compression and transmission of sensor data, the binary telemetry transport model (BTTM) was developed for the SPP.

#### 5.1.1. Binary Telemetry Transport Model

The binary telemetry transport model (BTTM) is a lightweight data model used to transfer sensor data between a sensor and the input component (i.e., a SourceNode) of the SPP. BTTM is mainly tailored to the requirements of a PLC and specifically developed in the context of the entire SPP development. Among others, it considers time synchronization issues and performance aspects with respect to the limited processing resources of a PLC. Note that BTTM offers no connection management like HTTP and is designed to transmit data as payload of common transfer protocols, like MQTT and AMQP.

**Figure 9:**
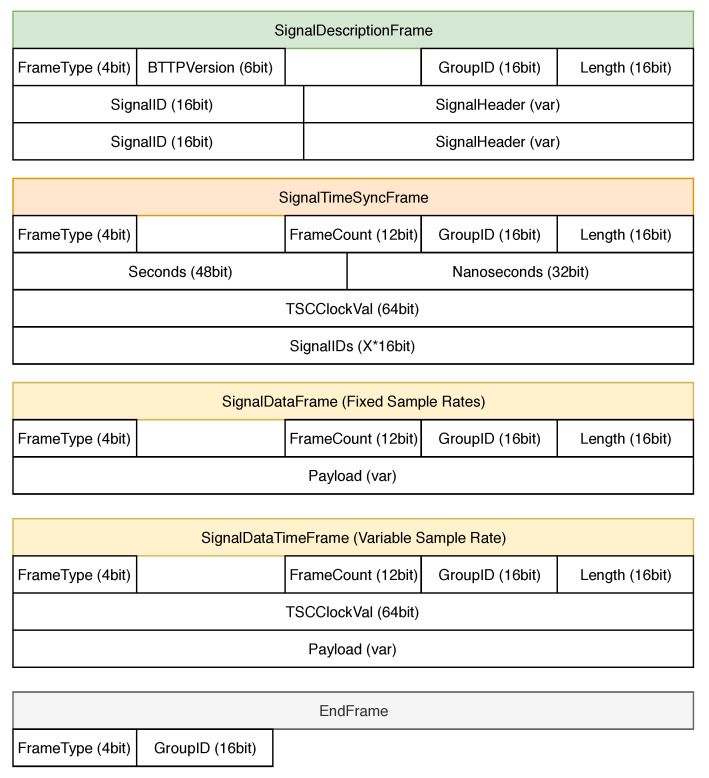
BTTM frames.

The BTTM is based on the artifact denoted with binary data frame, including various FrameTypes (see Figure 9). The frames carry signal meta-information and the sensor payload data. Every frame contains a fixed header including a 4-bit *FrameType* to distinguish the different frame types, i.e., *SignalDescriptionFrame*, *SignalTimeSyncFrame*, *SignalDataFrame*, and *EndFrame*. Different sensor signals, in turn, can be grouped by a *GroupID* header field. This approach allows for the transportation of frames from different groups concurrently. Finally, all frames contain a 16-bit length field to denote the end of a frame.

If a signal is transmitted via BTTM, its transmission has to be firstly initialized with a *SignalDescription* frame (see Figure 9). It contains a 6-bit BTTM version header, which must remain the same until a new *SignalDescription* frame is sent. *SignalDescription* frames contain key-value pairs with a 16-bit *SignalID* and a variable size *SignalHeader* field, which are used to transfer signal meta-information of transmitted signals of a group. For example, they may contain information about signal name, signal source, encoding, sample rate, and sample rate type in order to distinguish between fixed and variable sample rates, dead time, and a payload datatype. A *SignalDescription* frame is transferred whenever there is a change in the meta-information of a signal. For example, if a component of an Industry 4.0 machine is exchanged, a transmitted SignalDescription frame can denote this change. Note that each signal meta-information is valid until the next SignalDescription frame occurs.

After transmission of a *SignalDescription* frame, a *SignalTimeSync* frame is required in order to synchronize physical, and optionally, logical clocks such as a TSC between PLC and the SPP. Therefore, a *SignalTimeSync* frame consists of the standard header fields *FrameType*, *FrameCount*, *GroupID*, and *length*. In order to synchronize different system times, the Universal Coordinated Time (UTC) is used. Therefore, BTTM maintains a timestamp according to the IEEE1588 specification; i.e., it is mapped to a 48-bit seconds field and a 32-bit nanoseconds field, analogously to the Precision Time Protocol (PTP), which is widely used to synchronize time between manufacturing systems. IEEE1588 is designed to denote fractions of nanoseconds with an additional 16-bit field. Given different field bus systems, e.g., SERCOS, to capture sensor data, cycle times up to 31.25 ms are allowed. In practice, these cycle times are not used, as such precision cannot be provided due to limited PLC and bus-related transmission slot resources. Following this, BTTM does not provide the IEEE1588 16-bit nanoseconds field.

Optionally, a *SignalTimeSync* frame may contain a 64-bit *TSCClockVal* field to synchronize logical clocks. Thereby, one signal may be derived from one logical clock. Finally, in a *SignalIDs* field, related signal IDs are defined. Given a BTTM transmission with more than one signal, the latter may be related to different logical clocks within a group. This can be achieved by transmitting multiple *SignalTimeSync* frames, for which the *SignalIDs* field is set to the respective signals.

Sensor data, in turn, are transmitted through *SignalData* frames. In addition to specifying the *GroupID* that indicates the group membership, the length of the sensor data is specified in the 16-bit length field. Furthermore, a *SignalData* frame contains a variable size *Payload* field that contains the actual sensor data points. The payload format is variable and can be specified with a *SignalHeader* field in a *SignalDescription* frame.

For signals with a fixed sample rate, i.e., as specified in the *SignalDescription* frame, the correct timestamp of a transmitted data point can be calculated based on the *Seconds* and *Nanoseconds* fields, from the *SignalTimeSync* frame, and the specified sample rate.

For signals with a variable sample rate, either a *SignalTimeSync* frame has to be transmitted prior to the respective *SignalData* frame, or a *SignalDataTime* frame can be used. The latter is fundamentally the same as the SignalData frame. However, it contains an additional *TSCClockVal* field, which allows for an exact time per data point, without the need to transmit one *SignalTimeSync* frame for each SignalData frame.

The *End* frame indicates the end of the transmission of a group. Thereafter, no further frames of this group can be transmitted.

**Figure 10:**
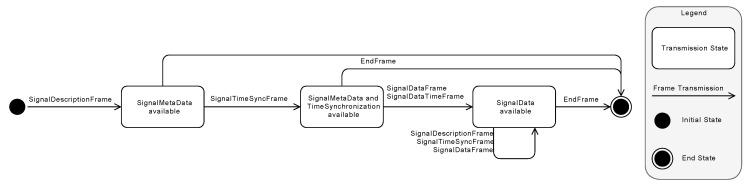
BTTM group state diagram.

The different BTTM transmission states are illustrated in Figure 10. A data stream is always starting with a *SignalDescription* frame, which describes the BTTM version and the signals to be transmitted; i.e., it defines the beginning of a group. Then, a *SignalTimeSync* frame follows, which allows a time synchronization for subsequent *SignalData* frames. *SignalDataTime* frames are a special feature as they contain a timestamp for a logical clock and thus allow calculations to be carried out quickly within a group. Finally, an **End** frame signals the end of a data stream.

**Figure 11:**
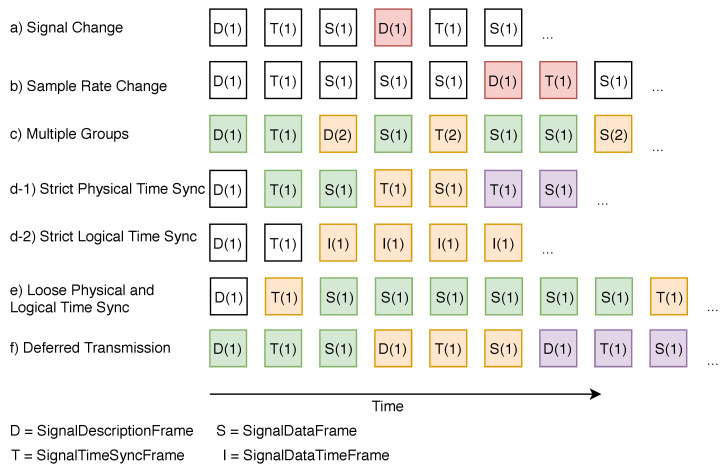
Examples of BTTM transmissions.

If a new signal shall be transmitted or the sample rate of a signal changes during transmission, a *SignalDescription* frame is transmitted containing the new signal information (see Figure 11a,b). With BTTM, multiple groups can be transmitted concurrently by setting different *GroupID*s for respective frames (see Figure 11c). If a *SignalData* frame has to be strictly time-synchronized (e.g., if a signal sample rate often changes or a timestamp must be assigned for each *SignalData* frame for efficient computation), each *SignalTimeSync* frame may follow one *SignalData* frame (see Figure 11d-1). If signals have to be strictly time-synchronized based on a logical clock, *SignalDataTime* frames can be used (see Figure 11d-2). Conversely, a *SignalTimeSync* frame may be used for several *SignalData* frames, if the sample rate does not change (see Figure 11e). If the transmission of signal data has to be deferred, for example, when acquired signal data are transferred with an unreliable connection and have to be buffered, the three frames to carry signal meta-information, time, and payload data can be grouped together (see Figure 11f).

In essence, BTTM is a lightweight sensor data transfer model enabling stream grouping and time-synchronization across different time bases to support streaming windowing and sensor data analysis, which are also fundamental pillars of the SPP.

### 5.2. Processing Pipeline Concept

**Figure 12:**
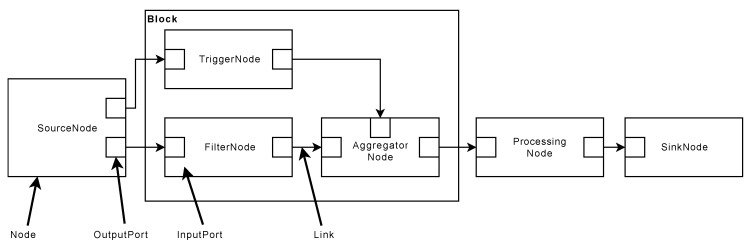
Processing pipeline concept.

When processing sensor data in the SPP, different steps have to be performed. For example, data serialization steps are needed, or an overall execution management must be governed. The SPP follows a flow-based approach to describe a processing pipeline that facilitates the flexible use of algorithms for signal processing [34]. The basic concept of processing stages through nodes is adopted (see Figure 12). Each node, in turn, is taking over specific processing step, which are explained in the following. Importantly, by linking individual nodes, complex processing sequences can be realized.

In contrast to information flow processing systems (IFP) [9], the SPP describes its internal control flow and data flow in a graph-oriented way, while data transformations are represented by node functions associated with every node in a processing pipeline. These concepts allow for the separation of data transformation rules, i.e., code implementations and their configuration. Following this, SPP node functions can be developed for multiple types of sensor data from different machines, while the SPP pipeline nevertheless supports the configuration of node functions for specific machines, which fosters reusability and the overall configuration management, including the management of variants. Furthermore, the SPP is conceived in a way that pipeline changes are possible, while relying on fixed node function sets on the other. For example, assume that an edge-based computing device (ECD) is directly connected to a machine, on which a SPP pipeline is deployed, and which is connected via an unreliable connection to the internet. In this case, changes to the SPP pipeline should be minimized in order to ensure comparability of the generated SPP results.

A SPP pipeline consists of nodes, ports, links, and blocks (see Figure 12). Each node encompasses zero or more input ports on one hand, and zero or more output ports on the other. Ports are used to describe data interfaces and in order to connect two nodes. Furthermore, the output port of one node is always linked to the input port of another node through the usage of links. Links, in turn, essentially describe the control and data flow between two nodes.

Incoming sensor data, denoted as “sensor signal,” is always converted by analog-to-digital (A/D) converters, resulting in sensor data values that are presented by discrete values (i.e., data points). Data points of a sensor, in turn, are transmitted with a fixed or variable sample rate; i.e., the time span between two incoming data points has either a fixed or variable length.

Production machines, including pharmaceutical packaging machines, consist of several devices, which typically run in a cyclic production process. In order to compare the different cycles, there must be a possibility to detect the cycle boundaries. The latter enable us to group data points of a cycle together.

Sensor signals shall be analyzed by the utilization of the SPP. Therefore, two scenarios must be distinguished: First, sensor signals should be processed in real time. The goal is to be able to react quickly to machine failures and prevent their possible damages. Note that real-time analyses imply the detection of machine problems without the need to apply sophisticated analysis methods [13]. Therefore, the provision and handling of real-time data is normally the desired way of handling signals. However, as the second scenario, there exist settings, in which sensor signals cannot be processed in real time. Here, usually, so-called batch processing strategies are utilized. In this case, sensor signals are analyzed afterwards by the use of sophisticated data science methods like machine learning [35,36]. However, the second scenario necessitates costly and time-consuming tasks. In order to address these two scenarios properly, SPP processing pipelines are always built on a synchronized aggregation stage and an asynchronous processing stage. During the synchronized aggregation stage, it is taken care of filtering sensor signals and normalizing and grouping of them (i.e., windowing). During the asynchronous processing stage, it is taken care of identifying windows that can be processed without delays and the threat of deadlocks.

Finally, a processing pipeline can comprise blocks, which provide the feature to group two or more nodes together. It is noteworthy that blocks follow the single-entry-single-exit (SESE) principle [37].

#### 5.2.1. Node Concept

**Figure 13:**
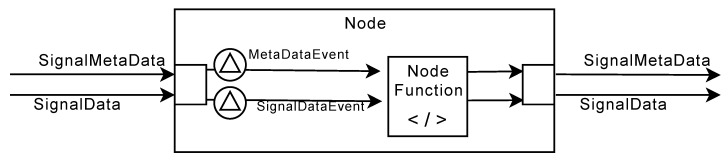
Schema of a SPP node.

The schematic structure of a node is shown in Figure 13. The SPP distinguishes between five different node types: A SourceNode, a FilterNode, a TriggerNode, an AggregatorNode for the synchronized aggregation stage, and a ProcessingNode and SinkNode for the asynchronous processing stage.

SourceNodes act as an interface for signal sources (e.g., PLCs). They serialize signals, and thus, only comprise OutputPorts. After the serialization procedure of a signal is finished, the signal is further processed. Thereby, FilterNodes, TriggerNodes, and AggregatorNodes are executed synchronously by accomplishing the following tasks: FilterNodes are used to transform and normalize signals, e.g., by applying band-pass filter algorithms. TriggerNodes, in turn, generate reference signals, which are then used by AggregatorNodes to apply windows to signals (see Section 5.3.2). The latter means that windowed signals are split into pieces, i.e., packages of two or more data points of a signal. These packages are then scheduled for processing through the ProcessingNodes. ProcessingNodes have at least one InputPort and one OutputPort. Finally, SinkNodes receive processed signal data points and persist or forward them to other subsystems, such as message brokers or specialized database systems.

Ports establish interfaces for exchanging data with other nodes. Exchanged data comprises windowed signal data points and associated signal meta-information. Thereby, every *NodeInput* and *NodeOutput* processes exactly one signal. Signals, in turn, consist of *WindowedData* and *MetaData* (see Section 5.6). Note that only a unidirectional communication is used, i.e., signal data points and signal meta-information is transferred only from a *NodeOutput* to a following *NodeInput*. A *NodeOutput* may have multiple connections to varying *NodeInputs*.

#### 5.2.2. Node Functions

**Figure 14:**
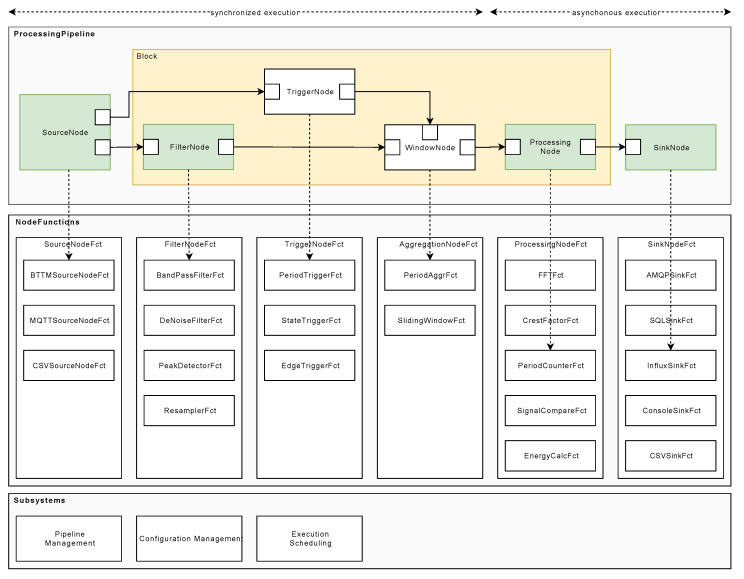
Overview of the sensor processing pipeline.

Nodes comprise executable components, i.e., NodeFunctions, which represent the executable program code that is eventually implementing the business logic of a node (see Figure 14).

The communication between *NodeInput* and a node function itself is defined by two events: The *WindowedDataReceived* event occurs whenever a new data element arrives at a node. In turn, the *MetadataChanged* event is called right before a *WindowedDataReceived* event, if and only if there is a new meta-information for a signal. The distribution as an event has the advantage that node functions are notified directly when new data are available for processing.

In addition to the data of the *NodeInputs* and *NodeOutputs*, reference data are managed by the node functions. Reference data, in turn, enables the feature to query valid contextual information (see Section 5.3.1).

*NodeOutputs*, in turn, receive metadata of other nodes and new *WindowedData* objects, and manage the connections to subsequent nodes. All incoming data objects are thus passed on to the following nodes.

### 5.3. Signal Aggregation

The source component of the SPP receives and processes data from various sensors. For this purpose, *SourceNodes* are conceived to be used with various transmission protocols. Standard IoT protocols, such as MQTT or AMQP, can be used to transmit data to the SPP with the presented BTTM model as payload.

SourceNodes take over the necessary communication with the sensors and convert sensor data supplied in various data formats if needed. For example, a conversion of the BTTM into a SPP-internal data model must be accomplished. Essentially, the internal data model must contain the following fields that are relevant for processing: The *SignalIdentifier* allows for the assignment of the measuring point to a signal. In addition, the measured value itself is stored in a *Value* field. The associated timestamp is specified in the field *Timestamp*. The allowed data types for the *Value* field are Doubles and Int32s. The *Timestamp*, in turn, can be expressed based on several time bases, but must be necessarily contain a real-time indication.

A *SourceNode* can receive data from multiple sensors and transmit them to following-up SPP nodes in a processing pipeline. In addition to sensor signal data, meta-information of the signals is captured as well, transferred to the internal data structure, and forwarded to the subsequent components. Signal meta-information is only transmitted if a change to the previous state has occurred. In addition to the *SignalIdentifier*, signal meta-information must contain information from which time on they are valid. In order to ensure the traceability of processing results in the SPP, the signal meta-information contains a list that records all processing stages along the processing pipeline.

Regarding performance aspects, each SourceNode can run in a separate thread. Furthermore, all following FilterNodes, TriggerNodes, and AggregatorNodes are executed in a separate thread. This allows for a fast preparation and distribution of sensor data without time-consuming context changes. To be more precise, incoming signal data can be buffered within a *SourceNode* and multiple signal data can be packaged into one *WindowedData* entity to enable faster serial processing in the following FilterNodes and TriggerNodes. *WindowedData* entities are exchanged between two threads with the help of Single-Producer/Single-Consumer (SPSC) queues and without the lock and monitor principle [38,39].

Afterwards, all transmitted signals can be filtered out by *FilterNodes* that are relevant and someone is interested in. Sensor data of the remaining signals are discarded. Optionally, further *FilterNodes* may take over pre-processing of the individual signals. This includes, for example, the smoothing of a signal by the formation of a moving average or a mathematical derivation for calculating the velocity from position data of a production machine. For efficient pre-processing, the following restrictions apply:

- Every *FilterNode* processes sensor data signals from the same *SourceNode* in order to show the same sampling time point.
- A *FilterNode* generates a signal of the same time resolution as the input signal; i.e., for every incoming data point exactly one output data point is generated and forwarded.

A combined processing of one or multiple signals and reference data correlation is executed in the following signal processing stage.

#### 5.3.1. Reference Data Management

In addition to sensor signal data and signal meta-information, data from other sources can be acquired (e.g., production data). These inputs are separately managed using the *ReferenceDataSources*. Depending on the implementation, different protocols such as MQTT or AMQP can be used. Therefore, relevant contextual information is parsed, transferred to the SPP internal structure, and finally passed to a *ReferenceDataController*. The internal representation is flexible but must contain at least one uniquely identifiable topic and a real-time stamp indicating whether this contextual information has changed. Within the *ReferenceDataController*, all *ReferenceDataSources* are merged. At the same time, all components that are interested in specific contextual information can register themselves and retrieve all information up to a certain point in time. This achieved observer pattern includes pull notifications unlike commonly used push notification concepts, and has the advantage that each component can selectively retrieve the contextual information at the time the information is actually needed; i.e., information from context and signal sources can be processed in a time synchronized manner. Consequently, incoming contextual information that does not have a registered customer/node is discarded.

#### 5.3.2. Windowing

**Figure 15:**
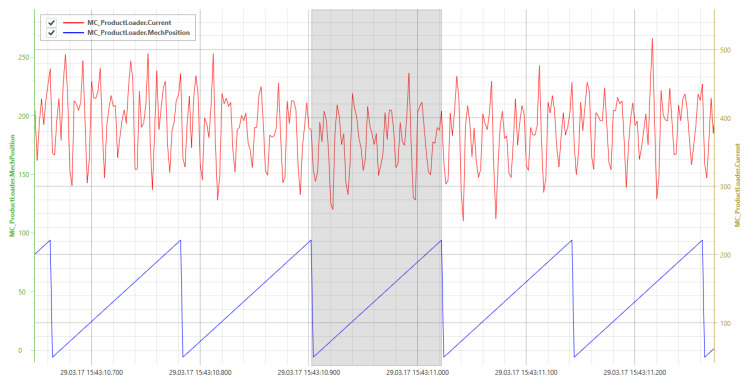
Correlated period of two sensor signals.

Prior to the actual processing of sensor signals, the conceived and implemented *windowing* has to be established. Note that the windowing splits incoming signals into windows for further processing and based on the time. Figure 15 shows an example of the data of two signals. In the upper area, a signal of a packaging machine is shown in red. Underneath, synchronously, the position signal of the motor is shown in blue. The position follows a cyclic process in this case, and thus, generates a sawtooth signal. Each cycle describes a process step within a machine and is therefore denoted as a period.

In the example shown in Figure 15, the position signal is used to determine the window boundaries, so that each process step of a machine is mapped to a window. Resulting windows are colored in gray. Since this window may depend on context data, e.g., the current speed of a machine, neither the number of measuring points nor the duration of the window is predefined.

Therefore, the concept of time windows or length windows known from complex event processing cannot be applied. Instead, other sensor signals or context data may influence the generation of windows. Therefore, the concept of *correlating windows* is used. Windowing in the SPP is done by two different node types: TriggerNodes and AggregatorNodes. *TriggerNodes* determine window borders. The respective extent of the *TriggerNode* determines whether *time windows*, *count windows*, or *correlating windows* occur. *AggregatorNodes*, in turn, combine the measurement points of a signal based on the window borders to *WindowedData* objects.

**Figure 16:**
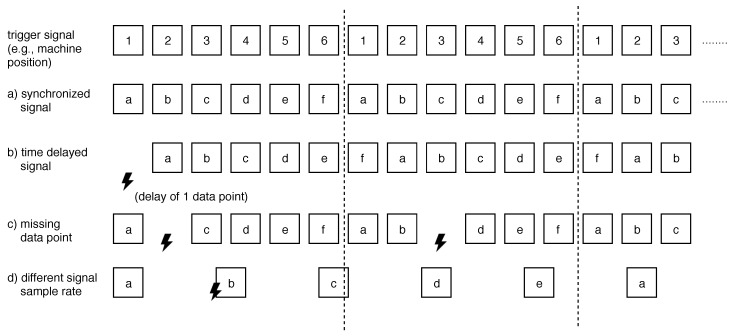
Data point synchronization.

During the aggregation of signal points, different challenges must be addressed: either data points from the signal to be windowed correlate perfectly with the generated or derived trigger signal (see Figure 16a). Or, there may be a time delay between the trigger and the signal to be windowed (see Figure 16b), which can be resolved by implementing a node function to buffer, and thus, synchronize the trigger signal. Furthermore, there may be missing data points, e.g., due to a sensor malfunction or transmission loss (see Figure 16c). In the latter cases, missing data points can be interpolated under some circumstances. Finally, trigger and window signals pose different signal sample rates (see Figure 16d), which can be solved by interpolations or triggering with an offset. To be more precise, current SPP *TriggerNode* implementations can be configured to send trigger events with a time offset.

### 5.4. Signal Processing

In the asynchronous signal processing stage, the actual processing of the sensor data is performed. Therefore, algorithms from various disciplines can be used. The SPP offers ProcessingNodes to execute a wide variety of algorithms. Each node takes over a single processing step, such as executing a Fast Fourier Transformation (FFT). By linking individual nodes, complex processing sequences can be realized.

Nodes and their components are generic; thus, signal meta-information, time bases, and WindowedData objects can be defined independently from the respective node function implementations. However, there are use cases, for which node functions must implement for a specific time base, which in turn, limits their general utilization.

#### Reference Data

Within nodes, it may be necessary to be able to access reference data depending on the algorithm used. To enable this, the SPP comprises interfaces with the persistence system presented below. In addition, it is possible to store arbitrary structured data in a memory for reference data and retrieving it easily by the use of a search feature, including the possibility to use search criteria. The basic structure of all reference data includes only a field for an unique ID and a real-time stamp indicating when the record was created. Depending on the application, different memories for reference data can be implemented and used. For example, an in-memory database can be used that stores reference datasets in the main memory.

### 5.5. Sinks

*SinkNodes* of the SPP are passing processed data streams to subsequent systems. These systems can have different tasks. For example, further processing and long-term comparisons can be carried out by means of machine learning, or textual or graphical outputs of the results can be generated. Due to this range of applications, the data protocols used are very differently. Conceptually, a *SinkNode* is a combination of a node without node output and a connector for the respective transmission protocol. Following this, results can be stored in databases, or it is possible to continue event-based data transportation. Therefore, common IoT protocols such as MQTT or AMQP can be used. Depending on the nature of the following system, it may be necessary to leave the processing in the thread pool through the *schedule controller* and use separate threads, which eventually take over the necessary communication.

### 5.6. Data Management

Various types of data exist when capturing, processing, and storing sensor data from a machine. The latter, for example, have different requirements for storage systems. Table 3 shows the different data types and their characteristics. Column “Schema” provides information on whether the data has a fixed payload format or differs between different applications. Column “Volume” records the amount of data that is generated depending on the data type. Column “Search Criteria” describes the characteristics for records that must be searchable. Column “retention period,” in turn, specifies whether the data records should be permanently stored in this data type or whether they can be deleted after a specific time period.

#### 5.6.1. Sensor Data

Incoming sensor data signals, e.g., from a PLC, have to be converted into a SPP-internal model. This is done by a SourceNode. Every signal within the SPP must consist of a *SignalIdentifier*, a *SignalMetaData*, a *WindowedData*, and payload data. The *SignalIdentifier* is converted into a hash, so that it can be compared more effectively to other SignalIdentifiers. *SignalMetaData* defines a *TimeBase* and a *TimeSpanBase*, which can be domain-specifically set for every incoming signal. Note that the processing nodes of the SPP pipeline are implemented with respect to a generic time base; i.e., they can process arbitrary time bases for different signals. *SignalMetaData* consists of a *SignalType* in order to define the type of the sample rate (i.e., “unknown”; “fixedsamplerate”; “variablesamplerate”), and a set of nodes, which a signal has been passed during its processing. Payload data of a signal is stored in a *DoubleRawDataItem*, consisting of a double value together with a SignalIdentifier and a generic time base. *WindowedData* defines a firstTime timestamp—for the beginning of the windowed signal, and a lastTime timestamp—for the definition of the end of a windowed signal. Again, both timestamps may be defined on a generic *TimeBase*.

#### 5.6.2. Reference Data

Reference data in the SPP is stored as key-value pairs in the *ReferenceDataController* and validated during SPP execution; i.e., they are used for temporary data. *NodeFunctions* can subscribe to relevant reference data updates based on the observer pattern.

#### 5.6.3. Result Data

Signal data, which was processed by the SPP pipeline, can be converted into a external model, e.g., to transfer them into a database. Therefore, a *SinkNode* must convert the signals.

#### 5.6.4. Pipeline Configuration Data

Common to all presented data types is, except for the pipeline configuration, that the stored data are not changed afterwards. For the pipeline configuration, new versions can result from parameter changes or new processing processes. However, the old pipeline configurations must be preserved for the traceability of stored results.

### 5.7. Pipeline Execution Management

In general, sensor signals from a manufacturing machine either have to be processed in real time or asynchronously. For example, to prevent a machine from self-damage, occurring errors have to be processed as fast as possible. On the other hand, there exist use cases, in which a noticeable larger amount of signal data can be processed batch-based, e.g., overnight. Therefore, the SPP is structured into two stages: aggregation and processing stage.

While nodes in the aggregation stage should run synchronously and as fast as possible, *ProcessingNodes* in the processing stage may be executed asynchronously.

**Figure 17:**
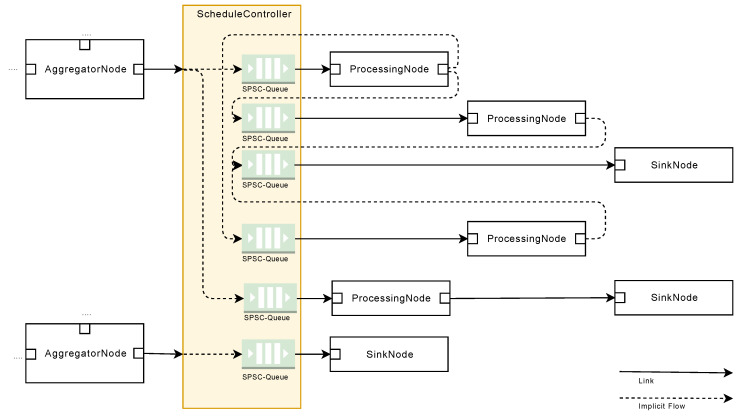
Schedule controller.

*ProcessingNodes* provide the basis for all computational-intensive processing tasks that run within the SPP. Therefore, *ProcessingNodes* are designed to run in parallel and multiple threads. The transition between *windowing* and parallel processing is realized by a *schedule controller* (see Figure 17). It manages the inputs of the first *ProcessingNode* (*HeadNode*) of each processing stage of a processing pipeline. In the *schedule controller*, a task list is created for each input of the HeadNodes. These tasks contain a *WindowedData* object for processing. In addition, the *schedule controller* has a configurable number of worker threads. These threads execute task lists, thus ensuring the execution of HeadNodes. During processing, the system schedules between *HeadNodes*, so that every processing line, starting from the *HeadNode*, is processed according to a round-robin scheduling method. The design of the *HeadNodes* scheduling method is also implemented via exchangeable implementations, i.e., suitable algorithms can be implemented depending on the application and the target system.

It is ensured that each *HeadNode* is executed by only one thread at a time as multiple worker threads handle *HeadNodes*. For this purpose, an efficient lock mechanism based on single-producer/single-consumer (SPSC) queues is implemented [38].

The execution of the further nodes of a processing pipeline always takes place on the basis of the current *HeadNode*. Since it must also be ensured that only one thread works in a node at a time, the following two cases must be distinguished: (1) nodes with exactly one *InputPort*, and (2) nodes with several *InputPorts*. Thus, if a node has only one *InputPort*, it has only one predecessor *Node*. Signal data are then passed in an event-based manner to a node, so that the executing thread changes from the ancestor *Node* to the following *Node*. As a consequence, only one node is executed in one thread at a time.

In order to realize nodes with multiple *InputPorts* and without any conflict, the *schedule controller* is used in combination with a *DoubleNodeInput*. The latter receives the *WindowedData* objects of the two predecessor nodes and stores them into two separate SPSC queues (see Figure 17). Since each node has its own list, no concurrency conflicts can occur. Then, a new task for editing a following node is created and stored in a SPSC queue in the *schedule controller*. Therefore, a real lock has to be used for the SPSC queue. Otherwise, both inputs of the *DoubleNodeInput* could write the latter at the same time. Since this task merely conveys the information that a new *WindowedData* object is ready for processing in *DoubleNodeInput*, no concurrency conflict can occur. The processing order of the tasks is irrelevant. Inside a *HeadNode*, there is a reference to the two lists in *DoubleNodeInput*. The node-specific implementation can retrieve and process the *WindowedData* objects from these lists according to the algorithm used. Notably, the concept presented for realizing a two-input node can be easily extended by adding new inputs.

## 6. Validation

### 6.1. Proof-of-Concept Implementation

In this section, the prototypical implementation of the SPP will be presented to demonstrate the technical feasibility of the concept. The proof of concept was realized in C# and is based on the .NET Framework from Microsoft. The implementation is structured into a domain-independent core package written in .NET Core, and a domain-dependent implementation written in .NET 4.6. The core implementation consists of a generic data model (including the considerations of time bases and data objects for signals and signal meta data), the core pipeline artifacts (interfaces and abstract implementation for all node types), and management interfaces. While the core package is written in .NET Core, it can be used in a system-independent manner (i.e., it runs on Windows and Linux systems).

The core implementation is extendable by a domain-specific implementation, i.e., comprises specific node implementations. Since the core implementation is separated from the domain-dependent implementation, the presented sensor data processing concept is generic and can be used for other applications. The presented prototypical implementation uses flexible data structures for sensor data, meta data, contextual information, and reference data.

Central data structures and superclasses are summarized in package *SPP.Core*. The latter constitutes the basis for all further projects. It is noteworthy that concrete implementations are not provided for the recipients; i.e., for *ProcessingNodes*, *WindowNodes*, *TriggerNodes*, or any other *node* no implementation templates are provided. They are realized in the domain-dependent projects. The latter contain all necessary data structures and implementations, which are especially needed for the processing of sensor data, e.g., for specific packaging machines. These include, for example, the *BTTMSourceNode* class, which reads PLC files in the BTTM format. The used generic types and their base classes are briefly introduced. In order to manage different time bases, the *SPP.Core* package contains the interface *TTimeBase*. It consists of a *RealTimeStamp* of type *DateTime*, and a *TTimeSpanBase*, which defines time spans in relation to the *TTimeBase*.

Sensor signal data are encapsulated into *TData* types, which define the data structure of the sensor signal data. Signal meta-information is represented by a *TMetadata* type, describing the structure of the metadata used for a specific sensor signal. Every node type used in the SPP, i.e., source, filter, trigger, aggregator, processing, and sink nodes, has a base class implementing the base features like the time synchronization. Every input and output of a node is implemented by a *NodeInput* class, and respectively a *NodeOutput* class. Context data can be registered in the *ContextController*, which acts as message-oriented middleware and offers topics to consuming processing nodes.

### 6.2. Visualization

**Figure 18:**
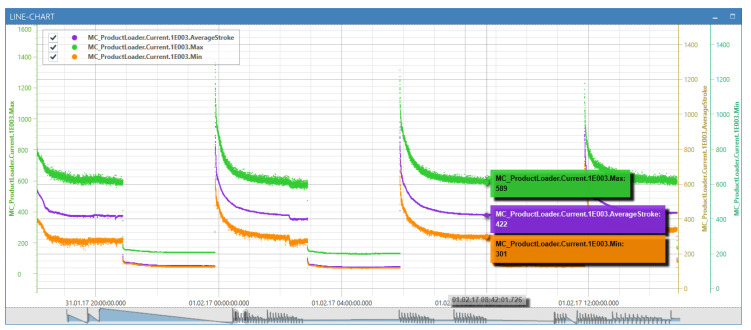
User interface of the SPP data visualizer.

In order to visualize the output of a SPP pipeline, a graphical interface was developed (see Figure 18). The interface is written in .NET as well and uses the SciChart (https://www.scichart.com) framework. SciChart is a graph framework, which is able to visualize a huge amount of data points in a single visualization by using Compute Unified Device Architecture (CUDA)-based rendering techniques [40]. We were able to visualize up to 52 million data points in a single graph close to real time, i.e., with a user interface response time of less than 100 ms. This corresponds to a visualization of 60 different sensor data sources with a resolution of a minute over a time of roughly 600 days.

### 6.3. Performance Analysis of the Sensor Processing Pipeline

The processing of the signals of a packaging machine must be able to be carried out in near real time to be able to react to changes as quickly as possible. On the basis of the performance analysis presented in the following, it is shown exemplarily that with the help of the SPP, 10 sensor data streams can be processed at 272 times of the real sensor data generation rate. Thus, sufficient performance reserves are available to process further data streams and to perform further analyses within the pipeline.

The sensor data of the case that has been shown had a resolution of 1ms and were transmitted using the BTTM. Typically, the resolution used for the application case is in the range of 5–10 ms, hence stricter assumptions were applied. The pipeline consists of processing nodes typical for the use case, including windowing and noise reduction.

#### Data Sources

For the performance analysis, records of a Uhlmann B1440 machine were used as the data source. These included a total of 10 signals that were sampled at a sampling point distance of 1 ms. The recording period was 44,101 s. All data was stored in the BTTM format, resulting in a total of 2566 MB.

#### Window Processing

The SPP processed two of the 10 signals, i.e., position and current power consumption of a B1440 packaging module. *SourceNodes* generated a new signal called *“CorrectedPosition,”* which is used to derive the correct position of an assembly group. The pipeline consisted of two parts, namely a statistical and a signal comparison part. The statistical part consisted of a statistical node (domain-dependent implementation of a *ProcessingNode*) to derive the average power consumption over 10,000 count windows. The node consists of 16 outputs for mean, median percentil, etc. Each result was stored in a database by a corresponding *SinkNode*.

The signal comparison part of the SPP pipeline consists of a *SawtoothTriggerNode* on the *“CorrectedPosition”* signal, an *AggregatorNode* based on *“CorrectedPosition”)* and current power consumption (based on the same trigger), a *NoiseCancelling-FilterNode*, which buffered windows and calculated mean values for noise reduction, a *signal comparison ProcessingNode* to compare noise cancelling results with reference data, and four *DatabaseSinkNodes*. Note that the *SawtoothTriggerNode* emits speed contextual information to the *ContextController*. The *NoiseCancelling-FilterNode* clears the buffer at significant speed changes. The signal compare node loads different reference data samples from a reference data store according to the current speed. In summary, 1 *FilterNode*, 1 *count TriggerNode*, 1 *correlating TriggerNode*, 3 *AggregatorNodes* (1 count, 2 correlating), and 2 *DatabaseSinkNodes* were created for performance analysis.

#### Execution Environment

The performance test was run on a laptop with a *Intel Core i7-6700HQ* CPU, a total of 8 logical processors with a maximum speed of 2.59 GHz, 16 GB of RAM, and a *SanDisk SD7TN3Q* SSD. Both files of the BTTM format and the data storage of the MongoDB were stored on the laptop. The processing was started with six worker threads, together with two *SourceNode* threads, all eight logical processors could be used.

#### Results

The processing was carried out three times, the values presented below are the resulting average values. The processing of all data was completed in an average of 162 s. This means, that the processing could be done at 272 times of the real sensor data generation rate. Referring to the input data amount, an input data stream of 15.8 MB/s could be processed. This corresponds to roughly 544,424 individual measuring points per second, which resulted in an usage of 85% of the available computational power. The remaining capacity was used by MongoDB and operating system processes.

## 7. Discussion

The SPP provides methods for capturing, processing, storing, and visualizing raw sensor data from sensor sources. A processing pipeline can be specified to describe the corresponding data processing workflow, similar to signal flow graphs, in a flexible way. For example, a sliding window node can be deployed to different pipelines, while sliding windows are generated differently based on pipeline-specific configurations. With the pipeline, sensor data can be retrieved from different protocols by using different *SourceNode* functions. The external data representation is mapped into the SPP-specific data model, which is a manual task. Future considerations will take automatic data conversion methods into account. Furthermore, the SPP is able to filter and pre-process sensor data before the data are windowed. Complex filter rule definitions are subject for future work.

With the SPP, node function implementations can be implemented independently from time bases and application data models, which are, for example, used by PLCs. Based on the SPP, we conducted another case study to detect anomalies in the power consumption of the product loader of a pharmaceutical packaging machine. For this purpose, SPP nodes for stream-based anomaly detection were implemented [13]. Although the development of an SPP node function is complex, the idea to develop them on a node basis enables the possibility to provide templates in future extension in a sophisticated repository [41].

Currently, the pipeline concept does not utilize conditional links. Conditional links can be used to determine, based on one or more sensor signals, whether successor nodes should be executed or not. However, this functionality can be implemented in node functions. In general, such conditions are only applicable based on signal and reference data. That means, there is currently no possibility to determine the execution state of different nodes beforehand to their execution. Currently, we are investigating possibilities to extend the SPP with a domain-specific language (DSL). Based on such extension, the SPP configuration could be defined in a more flexible way. A DSL enables the possibility for remote update calls, e.g., when the SPP is deployed to an IoT edge device, and adaptations regarding the processing should be deployed from a central service. The visualization component of the SPP currently supports a diagram-based visualization of data stored in an object-aware database. We want to extend the visualization component with an integrated development environment, in which developers and users are able to create new filtering, windowing, and processing algorithms, test them, and deploy new versions of a processing workflows to connected SPP instances. Finally, further improvements could comprise scheduling algorithms, which optimize the node execution based on the processed signal data. For example, such algorithms, together with a distributed execution service, could enable load-balancing of processing intensive nodes across a computing cluster.

Limitations of the framework are (1) a lack of runtime optimizations to improve computational efficiency, (2) missing technical concepts for a distributed node execution across system boundaries. Furthermore, although BTTM has been used and tested in real machine environments, (3) it has not been extensively evaluated, and (4) we assume that the transmission path between PLC and SPP is reliable and that probably missing data are interpolated and aggregated in the SPP. However, this fact does not pose a major problem if signal sample rates are chosen at large orders of magnitude.

To address the limitations, (1) the SPP can be developed in a different programming language and execution environment that manages hardware resources more efficiently than the implementation shown in C# and .NET. I/O operations play a major role in the execution of the SPP. For example, processing nodes can be optimized to require fewer I/O and CPU operations, such as array copy operations. Since the raised requirements do not require distributed execution, we have not considered limitation (2) in more detail, since this requires fundamental changes to the underlying execution semantics, e.g., the scheduling. We plan to address limitation (3) in a further publication with a detailed evaluation of BTTM. Since BTTM can be used as payload for standard IoT protocols, such as MQTT and AMQP, functions provided by the latter can be used to address limitation (4). For example, to address: transmission error detection and correction, quality of service parameters, transmission flow control, and integrity check functions.

## 8. Summary and Outlook

This paper introduced the concept and a prototypical implementation of a sensor processing pipeline (SPP). The latter provides methods for sensor data capturing, transmission, processing, and storage. The SPP uses a flexible graph-based pipeline concept to process incoming sensor data. In addition, it is possible to integrate contextual information from separate sources into the SPP in order to use data that can be compared for sensor data processing.

A generic proof-of-concept implementation shows the feasibility of the concept. The SPP was developed to process sensor data of pharmaceutical packaging machines, but can be also applied to arbitrary discrete sensor signal streams. To efficiently transmit and store sensor data, e.g., between a PLC and the SPP, the BTTM model has been defined. By using this protocol, sensor data with different sampling rates, different time bases, and the associated metadata can be transmitted in an efficient way.

In future work, we deal with the development of a domain-specific language for sensor processing pipelines in order to declaratively define a processing workflow [42], and be able to create an IoT edge computing infrastructure, in which huge amounts of raw sensor data can be pre-processed and transmitted to cloud environments [43]. Furthermore, the visualization for the processing pipeline shall be extended in a way that a developer is able to create and manage processing workflows visually by using an integrated development environment. Finally, the selection of scheduling algorithms based on signal data shall be improved in order to balance the execution of the SPP pipeline for processing intensive nodes in parallel. Altogether, especially in Industry 4.0 settings, the processing of sensor data becomes more and more important. However, complex frameworks are needed to properly cope with the sheer size of sensor data that are daily generated daily. We consider the concepts shown with the SPP as a first promising starting point for future development directions.

## Figures and Tables

**Table 1:** Sensor data processing requirements.

	SPP	Apache Spark	Apache Flink	Apache Storm	Dunkel et al.	Borealis	LWDF	FastFlow	KNIME	Schweppe et al.
R1: Stream processing	X	X	X	X	X	X	X	X	- *^a^*	X
R2: Variability support (machine/sensor variants)	X *^b^*	(X) *^c^*	(X) *^c^*	X *^d^*	?	?	(X) *^e^*	(X) *^e^*	(X) *^f^*	(X) *^c^*
R3: Support for (non-)equidistant data streams	X	X	X	X	?	?	-	X	X	?
R4: Data model transformations	X	X	X	X	?	?	-	X	X	X
R5: Gap detection/interpolation	X/X	X/X	X/X	X/X	?/?	X/X	X/-	X/X	X/X	-/-
R6: Parallel processing	X	X	X	X	X	X	-	X	X	X
R7: Flexible time bases for sensor data	X	-	-	-	-	-	-	-	-	-
R8: Grouping of streams	X	X	X	X	-	?	-	-	-	-
R9: Time synchronization of signals in a group	X (relative, absolute *^g^*)	-	-	-	-	-	-	-	-	-
R10: Windowing based on machine speed *^h^*	X	-	-	-	-	-	-	-	-	-
R11: Windowing trigger definition usable for multiple streams	X	-	-	-	n/a	-	-	-	-	-
R12: Commodity hardware support	X	-	-	-	n/a	(X)	X	X	-	X

X = supported, (X) = partly supported, - = not supported, n/a = not applicable, ? = no information available; SPP = sensor processing pipeline, LWDF = lightweight dataflow, KNIME = Konstanz Information Miner; *^a^* stream processing is under development; *^b^* pipeline for a set of variants; *^c^* query per variant; *^d^* via TopologyBuilder; *^e^* flow per variant; *^f^* pipeline per variant; *^g^* with BTTM—see Section 5.1.1; *^h^* windowing adaption across multiple streams calculated based on other streams (correlated windowing).

**Table 2:** Overview of discussed aspects and goals.

Discussed Aspects	
**Aspect**	**Importance**
Uhlmann Pac-Systeme GmbH and Co. KG	The case study presented in Section 3 was developed in cooperation with Uhlmann on the basis of pharmaceutical packaging machines developed by them.
Programmable logic controller	Acts as a data source for production systems.
Time management in PLCs	Precise clock synchronization across different systems is required to correlate distributed data.
Information flow processing	To be able to use a large amount of sensor data in downstream systems, they must be converted, normalized and interpreted into standardized data models.
Data stream windows	PLCs provide continuous data streams. In order to be able to correlate and analyze them, these data streams must be divided into individual data packets with the help of windowing. This procedure is not trivial and requires application-specific customization.
**Goals**	
**Goal**	**Description**
Context-aware Process Management	In order to enable business processes to process and interpret events generated by business processes, custom concepts are required for the integration and processing of these events within process aware information systems.
Context-aware Process Injection	Cyber-physical systems are subject to constant state changes, which can also have an impact on business processes. In order to implement changes in already running process instances, we introduce the concept of context-aware process injection (CaPI).

**Table 3:** Data Types and their Properties.

Data Type (Schema)	Volume	Search Criteria	Retention
Sensor Data (fixed)	high	timestamp	flexible
Reference Data (flexible)	low	freely selectable	flexible
Results Data (flexible)	medium	timestamp	flexible
Pipeline Configuration (fixed)	low	none	permanent

## Abbreviations

The following abbreviations are used in this manuscript:

BTTM	Binary Telemetry Transport Model
CAN	Controller Area Network
CEP	Complex Event Processing
FFT	Fast Fourier Transformation
IoT	Internet of Things
PLC	Programmable Logic Controller
PTP	Precision Time Protocol
RTC	Real-Time Clock
SPP	Sensor Processing Pipeline
SESE	Single-Entry-Single-Exit
SPSC	Single-Producer/Single-Consumer
TSC	Time Stamp Counter

## Appendix A. Overview of Data Processing Frameworks

**Table A1:** General Aspects.

	SPP	Apache Spark	Apache Flink	Apache Storm	Borealis	FastFlow	KNIME	Schweppe et al.
Intended use	Sensor data processing of synchronized PLC-based machines/systems	Framework for cluster computing	Streamprocessor- framework	Streamprocessor- framework	Streamprocessor- framework	C++ parallel programming framework	Data analytics platform	Automotive diagnosis data analysis (OBD + CAN)
Target runtime environment (R12)	Commodity hardware	Distributed datacenter environment (Hadoop cluster)	Distributed datacenter environment	Distributed datacenter environment	Commodity/ datacenter	Commodity	Commodity/ datacenter	Embedded controller
Required RAM (R12)	512 MB (.NET Framework) + 50 MB (SPP) + node implementations + processing data = 562 MB++	Single: 8 GB (recommended) + node implementations + processing data	Single: 4 GB (minimum), 8 GB (recommended) + node implementations + processing data	Single: 8 GB (recommended) Cluster: 2 Nimbus nodes (2 × 8 GB), 2 worker nodes (2 × 8 GB) = 32 GB++ (recommended)	2 GB per node	?	8 GB (recommended)	< 128 MB

**Table A2:** Functional Requirements.

	SPP	Apache Spark	Apache Flink	Apache Storm	Borealis	FastFlow	KNIME	Schweppe et al.
**Processing Type**	Hybrid	Hybrid	Hybrid	Stream	Stream	Stream	Batch ^1^	Stream
Stream processing (R1 and SR1)	X	X	X (via DataStream API)	X	X	X	-	X
Stream processing type	Native	Micro-batching	Native	Native	Native	Native	n/a	Native
Batch processing	X (via stream replaying)	X	X (via DataSet API)	-	-	-	X	-
X = supported, (X) = partly supported, - = not supported, n/a = not applicable, ? = no information available								
SPP = sensor processing pipeline, LWDF = LightWeight DataFlow, KNIME = KoNstanz Information MinEr								
**Execution Model**								
Execution model described as directed acyclic graph	X	X	X	X	- (implicit via QueryProcessor)	X	X	X
Explicit execution model	X (pipeline)	-	-	X (via TopologyBuilder)	- (implicit via QueryProcessor)	X (blocks, pipelines, farms)	X (pipeline)	X (stream processing graph)
Dedicated data processing	X (source, sink)	X (input, output)	X (source, sink)	X (spouts)	X	X (input, output)	X (reader, writer)	X (source, sink)
Data queuing to decouple data generation and processing	X (SPSC FIFO)	X	X	X	?	X (SPSC FIFO)	X	?
Query language support (SR2)	-	(X)	X (via Table API)	X	X (SQuAl)	-	(X) (SQL)	X (DeviceSQL)
Query planning	n/a	X	?	?	X	n/a	-	?
**Data Processing**								
Processing components	X (nodes, stateful/stateless)	X	X (nodes, stateful/stateless)	X (bolts, stateless)	X (boxes, stateless)	X (worker, stateful/stateless)	X (nodes, stateless)	X (innernodes, stateless)
Datastream transformations (R4)	map, filter, partition, reduce, aggregate, windowing, split (stream), join (window)	map, filter, transform, repartition, reduce, union (stream), windowing, split (stream), join (stream)	map, filter, partition, reduce, fold, aggregate, windowing, union (stream), split (stream), join (window, interval), physical partitioning	map, filter, repartition, reduce, fold, aggregate, windowing, branch, union (stream), split (stream)	map, filter, union, bsort, aggregate, join (window), resample	map, filter, reduce, stencil, farm, split, join, ...	map, filter, reduce, fold, aggregate, branch, union, split	filter, windowing
Gap detection/ interpolation (R5)	X/X	X/X	X/X	X/X	X/X	X/X	X/X	-/-
Serialization required between Processing Nodes	-	-	-	-	n/a	-	X (conversion to data table)	-
State management	stateful/stateless	stateless	stateful/stateless	stateless/ stateful (via Trident)	stateless	stateful/stateless	stateful	stateless
Stream meta-information	X	X	X	X	?	-	n/a	?
**Windowing**								
Windowing support	X (sliding, tumbling)	X (sliding, tumbling)	X (sliding, tumbling, global ^2^)	X (sliding, tumbling)	X (sliding, tumbling)	X (sliding, tumbling)	-	X (sliding, tumbling)
Runtime windowing adaption (R10)	X (based on other streams, i.e., correlating)	-	-	-	-	-	n/a	-
Window trigger	time (flexible), count (flexible), correlating	time (fixed), count (flexible)	time (fixed), count (flexible), session	time (fixed), count (fixed), session	time (fixed), count (flexible), session	time (fixed), count (flexible)	n/a	time, count
Explicit window trigger nodes (EWTN)	X	-	X	(X) (via TriggerPolicy)	X (distinct box types)	-	n/a	-
EWTN for different streams (R11) ^3^	X	-	-	-	-	-	n/a	-
Flexible time base for windowing/ processing (R7)	X (e.g., relative TSC, absolute RTC, IEEE1588 ns precision)	- (absolute unix_timestamp or duration, μs precision)	- (absolute unix_timestamp or duration, ms precision)	- (absolute duration, ms precision)	-	-	n/a	-
Allowed lateness ^4^	-	-	X	-	-	-	n/a	-
**Customization**								
Custom processing	X (custom nodes)	X (User-defined Functions UDF)	X (functions, operators)	X (via Trident)	- (8 operators)	?	X (repository with over 1500 nodes)	?
Parameterizable pipeline templates (R2)	X	-	-	-	-	X	X	X
Contextual data (SR5)	X (Key-value)	(X) (Streaming Context)	X (Tables)	X (Trident)	?	?	X	-
TSC = Time Stamp Counter, RTC = Real Time Clock								

^1^ Stream processing is under development; ^2^ Assigns data frames with the same id to the same window, must be used with window trigger; ^3^ Enables to use a window trigger for multiple window generators for different streams; ^4^ Data belonging to a window is not dropped, if the former arrives after window generation.

**Table A3:** Non-Functional Requirements.

	SPP	Apache Spark	Apache Flink	Apache Storm	Borealis	FastFlow	KNIME	Schweppe et al.
Robustness (SR4)	X	X	X	X	X	X	X	?
Low latency (SR8)	X (when not applying compute-intensive algorithms in the asynchronous processing part)	no true streaming, not suitable for low latency	X	X	?	X	-	X
Delivery guarantees	exactly once	exactly once	exactly once, atleast once ^1^	atleast once	atleast once	exactly once	n/a	exactly once
Error correction (SR3)	X (detection and correction/ interpolation of missing frames)	(X) (manual implementation)	X	X (detection and correction/ interpolation of missing frames)	X	(X) (manual implementation)	-	?
Extensibility	Data sources, processing, data sinks	data sources, processing, data sinks	Data Sources, processing, data sinks	data sources, processing, data sinks	?	X	X	?
Availability (SR6) ^2^	-	X (distributed setup)	X (distributed setup)	X (distributed setup)	X (distributed setup)	-	-	-
Scalability (SR7)	- (vertically)	X (horizontally)	X (horizontally)	X (horizontally)	X (horizontally)	- (vertically)	X (horizontally via Hadoop)	- (vertically)

^1^ Configurable—dataflows with parallel streaming operations, e.g., map, flatMap, filter, are "exactly once," even in "atleast once" mode; ^2^ Operational system monitoring, measurement, and management.
